# *SYL3-k* increases style length and yield of F_1_ seeds via enhancement of endogenous GA_4_ content in *Oryza sativa* L. pistils

**DOI:** 10.1007/s00122-021-03968-y

**Published:** 2021-10-17

**Authors:** Xiaojing Dang, Yuanqing Zhang, Yulong Li, Siqi Chen, Erbao Liu, Bingjie Fang, Qiangming Liu, Dong She, Zhiyao Dong, Zhilan Fan, Dalu Li, Hui Wang, Shangshang Zhu, Xiaoxiao Hu, Yanhui Li, Jianhua Jiang, Delin Hong

**Affiliations:** 1 State Key Laboratory of Crop Genetics and Germplasm Enhancement, Nanjing Agricultural University, Nanjing, 210095 China; 2 Institute of Rice Research, Anhui Academy of Agricultural Sciences, Hefei, 230031 China; 3 Special Crop Research Institute, Chongqing Academy of Agricultural Sciences, Chongqing, 402160 China; 4 Department of Student Affairs, Jilin Normal University, Siping, 136000 Jilin China; 5 Rice Research Institute, Guangdong Academy of Agricultural Sciences, Guangzhou, 510640 China; 6 School of Agriculture and Biology, Shanghai Jiao Tong University, Shanghai, 200240 China

## Abstract

**Key message:**

*SYL3-k* allele increases the outcrossing rate of male sterile line and the yield of hybrid F_1_ seeds via enhancement of endogenous GA_4_ content in *Oryza sativa* L. pistils. The change in style length might be an adaptation of rice cultivation from south to north in the northern hemisphere.

**Abstract:**

The style length (SYL) in rice is one of the major factors influencing the stigma exertion, which affects the outcross rate of male sterile line and the yield of hybrid F_1_ seeds. However, the biological mechanisms underlying SYL elongation remain elusive. Here, we report a map-based cloning and characterisation of the allele *qSYL3-k*. The *qSYL3-k* allele encodes a MADS-box family transcription factor, and it is expressed in various rice organs. The *qSYL3-k* allele increases SYL via the elongation of cell length in the style, which is associated with a higher GA_4_ content in the pistil. The expression level of *OsGA3ox2* in pistils with *qSYL3-k* alleles is significantly higher than that in pistils with *qSYL3-n* allele on the same genome background of Nipponbare. The yield of F_1_ seeds harvested from plants with 7001S^*SYL3−k*^ alleles was 16% higher than that from plants with 7001S^*SYL3−n*^ allele. The sequence data at the *qSYL3* locus in 136 accessions showed that alleles containing the haplotypes *qSYL3*^*AA*^, *qSYL3*^*AG*^, and *qSYL3*^*GA*^ increased SYL, whereas those containing the haplotype *qSYL3*^*GG*^ decreased it. The frequency of the haplotype *qSYL3*^*GG*^ increases gradually from the south to north in the northern hemisphere. These findings will facilitate improvement in SYL and yield of F_1_ seeds henceforward.

**Supplementary Information:**

The online version contains supplementary material available at 10.1007/s00122-021-03968-y.

## Introduction

Rice (*Oryza sativa* L.) is one of the important cereal crops and is consumed by 3.5 billion people worldwide. With an increase in the human population and the decrease in the effective area for food production, it is imperative to raise the yield of rice per unit area to ensure food security for millions of people globally in the near future. Cultivating hybrid varieties of rice is an effective and feasible strategy to enhance the rice grain yield. Hybrid rice cultivars out yield conventional rice cultivars by approximately 20% (Lu & Hong 1999; Cheng et al. 2004). However, the annual production of fresh F_1_ hybrid seeds is required for large-scale hybrid rice cultivation. The area and yield of hybrid F_1_ seeds in previous seasons determine the acreage of hybrid rice planting for the next season. For a given amount of spikelets per unit area, the yield of F_1_ hybrid rice seeds is largely determined by the outcrossing seed setting rate, which is mainly affected by the stigma exertion. Increasing the percentage of exerted stigmas (PES) of male sterile lines (used as the female parent in F_1_ seed production in the field) could increase the chance of receiving pollen after flowering, which compensates for the poor pollination rate caused by parental differences in the flowering time (Wang et al. 2008). Additionally, there was a remarkable positive correlation between the PES and F_1_ hybrid seed yield in a study, with an increase of 1% in the PES of male sterile lines, 0.74–0.92% in the seed setting rate, and 47–68 kg/hectare in the seed yield (Yang 1997). The PES is largely determined by the stigma length (STL), style length (SYL), and the sum of the stigma and style length (TSSL).

In rice, the stigma characteristics, a complex quantitative trait, are controlled by multiple genes with minor phenotypic effects (Uga et al. 2003, 2010; Yu et al. 2003). To the best of our knowledge, 37 quantitative trait loci (QTLs) have been detected for the STL and they are distributed on all the 12 chromosomes (Uga et al. 2003, 2010; Yan et al. 2009; Li et al. 2010; Marathi et al. 2015; Dang et al. 2016, 2020). Uga et al. (2003) reported that the QTL *qSTL-4* on the chromosome 4 has the largest additive effect, and the allele from accession W1944 can increase the STL by 0.106 mm compared with Pei-kul. Thirty-nine QTLs have been identified for the SYL and they are located on chromosomes 1, 2, 3, 4, 6, 7, 9, 10, and 11 (Uga et al. 2003, 2010; Li et al. 2010; Marathi et al. 2015; Dang et al. 2016, 2020; Zhou et al. 2017). Uga et al. (2010) also reported that the QTL *qSYL-3* on the chromosome 3 has the largest additive effect, and the allele from IR64 can increase the SYL by 0.102 mm compared with Kinandang Patong. Finally, 30 QTLs have been identified for the TSSL and they are distributed on chromosomes 1, 2, 3, 4, 6, 7, 9, and 12 (Li et al. 2010; Liu et al. 2015a; Marathi et al. 2015; Dang et al. 2016, 2020). The percentages of phenotypic variance explained by these QTLs for TSSL range from 2.9 to 20.0%. Li et al. (2010) reported the QTL *qSSL-3* (SSL = TSSL here) on the chromosome 3 has the largest additive effect, and the allele from accession T821B can increase TSSL by 0.092 mm compared with G46B. Among all these QTLs, one candidate gene, *LOC_Os03g14850* (originally named *qSTL3*), has been identified for controlling TSSL (Liu et al. 2015b). Two genes, *OsSYL2* and *OsSYL3*, were identified for controlling the SYL (Dang et al. 2020). However, the underlying mechanism of action of these genes is still unclear.

In our previous study, a major QTL, *qSTL3*, was finely mapped to a 19.8-kb region in the short arm of the chromosome 3, and the *LOC_Os03g14850* gene was validated as the gene of *STL3* (Liu et al. 2015b). Because the STL reported by Liu et al. (2015b) actually included the total length of two parts (stigma and style) of the pistil, and no significant phenotypic difference in the STL was found between Nipponbare allele and Kasalath allele at this gene locus, we renamed the original QTL *qSTL3* as *qSYL3* in the present study to correctly reflect the real difference. In this study, we further verified that *LOC_Os03g14850* is the *SYL3* gene using transgenic complementation and overexpression experiments and determined the biological functions of the *SYL3-k* allele from the rice cultivar Kasalath. We also evaluated the potential importance of the *SYL3-k* allele in hybrid rice seed production utilising field experiments and elucidated the allelic polymorphism at the *SYL3* locus in 136 accessions collected from different geographic zones.

## Materials and methods

### Rice materials and field planting

Rice cultivars Nipponbare, Kasalath, SSSL14, and 101 accessions in the core collection of rice were cultivated in a paddy field in Nanjing (118.6° E, 32.1° N). Thirty-five wild rice accessions were cultivated at the National Germplasm Guangzhou Wild Rice Nursery in Guangzhou (113.2° E, 23.1° N). The information of these accessions, including the name, latitude, and longitude, is listed in Supplementary Table S1. Rice cultivar SSSL14 is a single-segment substitution line with the genome background of Nipponbare and contains only one donor fragment from Kasalath in the middle of the short arm of chromosome 3. The transgenic plants were grown in the conditions prevalent at Nanjing and Hainan (110.0 °E, 18.5 °N). On the field, the rows were spaced 20 cm apart, and each plant was spaced at a distance of 17 cm according to standard agronomic management practices.

### Trait measurement

At the heading stage, 10 target spikelets were collected from five plants of each accession prior to glume opening (at approximately 10:00 a.m.). For each spikelet, the STL, SYL, and TSSL of the fertile floret were measured using a stereo microscope (10 ×, MC50, Guangzhou, China), and the average values (in mm) of the 10 florets were taken for the accessions.

### Sample preparation and observation under light microscopy

In order to investigate the cytologic reasons that affect the SYL, we conducted sheet pressing analysis using the styles of Nipponbare, SSSL14, Kasalath, transgenic complementation plants, and overexpression plants. At anthesis, the mature pistils of Nipponbare, SSSL14, Kasalath, and transgenic positive plants (including the complementation and overexpression plants) were observed under a light microscope (Olympus FV1000, Japan). The pistils were then sampled and fixed with 70% (v/v) alcohol, 38% (w/v) formaldehyde, and glacial acetic acid (90:5:5, by volume), washed with tap water three times, and placed in 5 μl of 6% NaOH between a cover glass and a microscope slide and a pressed plate. Longitudinal sections were then observed under a light microscope.

### Application of exogenous phytohormones on the top second leaf blades of main stems

During the third or fourth stage of young panicle differentiation (according to the criteria reported by Itoh et al. (2005)), the second leaf blades from the top of the main stem of different Nipponbare and SSSL14 plants were smeared with 10 μM gibberellic acid (GA_3_; Shanghai Ryon Biotechnology Co., Ltd, Shanghai, China), 20 μM brassinosteroid (BR; Sigma-Aldrich Trading Company Ltd, Shanghai, China), and 1 μM indole-3-acetic acid (IAA; Beijing Solarbio Science & Technology Co., Ltd, Beijing, China) using a hygroscopic cotton ball. Control plants were treated with absorbent cottons containing the same volume of distilled water. The smear concentrations of GA_3_, BR, and IAA were determined according to a previous report (Zhao et al. 2010). Finally, we sampled the spikelets to measure the STL, SYL, and TSSL at the flowering stage.

### Endogenous phytohormone content analysis using high performance liquid chromatography-tandem mass spectrometry (HPLC–MS/MS)

The content of endogenous plant hormones GA_1_, GA_4_, BR, and IAA in mature pistils at stage 8 of young panicle differentiation in the Nipponbare and SSSL14 plants was detected utilising the methods reported by Durgbanshi et al. (2005), Forcat et al. (2008) and Zhong et al. (2013).

The phytohormone content of GA_1,_ GA_4_, and IAA was measured using HPLC (1260, Agilent technologies, USA) and MS (6420A, Agilent technologies, USA). The chromatographic conditions were: Poroshell 120-SB-C18 reversed phase column (150 × 2.1 mm, 2.7 μm); column temperature: 30℃; sample size: 2 μl; mobile phase: *A*:*B* = (methanol/0.1% formic acid): (water/0.1% formic acid); flow velocity: 0.3 ml/min; and gradient elution mode: 0–1 min, 20% *A*; 1–3 min, 20–50% *A*; 3–9 min, 50–80% *A*; 9–10.5 min, 80% *A*; 10.5–10.6 min, 80–20% *A*; 10.6–13.6 min, 20% *A*. The mass spectrometry conditions were: ESI^−^ negative ion mode; scan type: multiple-reaction monitoring; air curtain: 15 psi; spray voltage: − 4000 v; atomising pressure: 65 psi; auxiliary pressure: 70 psi; and atomisation temperature: 400 °C.

The BR content was measured using HPLC (1290, Agilent technologies, USA) and tandem MS (SCIEX-6500 Qtrap, Allen-Bradley, USA). The chromatographic conditions were: Poroshell 120-SB-C18 reversed phase column (150 × 2.1 mm, 2.7 μm); column temperature: 35 °C; sample size: 2 μl; mobile phase: *A*:*B* = (methanol): (water/0.1% ammonia); flow velocity: 0.35 ml/min; and gradient elution mode: 0–2 min, 80% A; 2–3.5 min, 80–95% A; 3.5–6 min, 95% A; 6–6.1 min, 95–80% A; 6.1–10 min, 80% A. The mass spectrometry conditions were: ESI^−^ positive ion mode; scan type: multiple-reaction monitoring; air curtain: 15 psi; spray voltage: + 4500 v; atomising pressure: 65 psi; auxiliary pressure: 70 psi; and atomisation temperature: 350 ℃.

For the calculation of hormone content in the samples, we used the following formula: hormone content in the sample (ng/g fresh weight) = detection concentration (ng/ml) × volume coefficient (ml)/mass coefficient (g), where the volume coefficient is the volume of the solution used in the final dissolution of the sample and the mass coefficient is the mass of the sample.

### Plasmid construction and plant transformation

To produce the complementation construct p*CAMIA1300*-*SYL3*, a 4844-kb *SYL3* genomic DNA fragment including the 2221-kb upstream sequence, full-length 2223-kb *SYL3* sequence, and 400-kb downstream sequence was amplified from Kasalath and cloned into the plant binary vector p*CAMBIA1300* using In-Fusion™ Advantage PCR Cloning Kits (Takara, Japan). To generate the overexpression constructs, the full-length coding sequence (CDS) of *SYL3* was amplified from Kasalath and cloned into the plant binary vectors p*BWA(V)HS* with a 35S promoter. A 2321-kb DNA fragment upstream of the *SYL3* start codon was amplified from Kasalath and cloned into the p*BWD(LB)1C*–GUS plus vector to generate the plasmid *PRO*_*SYL3*_:*GUS*.

We used the site-directed mutation method to construct two single nucleotide polymorphism (SNP) constructs (H2 and H3, the allele-specific transformation vectors). Firstly, the *SYL3-n* CDS was used as the template to be modified by site-directed mutagenesis based on the primers containing the mutated nucleotides. For the H2 vector, the base G in position S1 was replaced by the base A. For the H3 vector, the base G in position S2 was replaced by the base A. The PCR products were cloned into the plasmid p*BWA(V)BS* using the homologous recombination method. The genotype of SSSL14 is H1, and the genotype of Nipponbare is H4. All the site-directed mutagenesis transgenes were driven by the *SYL3-n* promoter. The primer sequences used for the vector construct are listed in Supplementary Table S2.

We verified all the resultant constructs by sequencing, and then, the binary vectors were electroporated into the *Agrobacterium tumefaciens* strain EHA105 and transformed into Nipponbare (e.g. empty vector, complementation vector, *pSYL3-k*::GUS, H2, and H3), SSSL14 (e.g. empty vector and overexpression vector), and 7001S (e.g. complementation vector) using the method described by Hiei et al. (1994). Positive *pSYL3-k*::GUS transgenic plants were selected based on the antibiotic resistance, and GUS histochemical staining analysis was conducted using the method described by Jefferson et al. (1987). Images were captured using a stereo light microscope (Leica DFC 420, Leica Microsystems, Germany).

### Subcellular localisation

The SYL3-k-RFP in-frame fusion protein construct and the nuclear marker NLS-mCherry construct were co-expressed transiently in rice leaf protoplasts using the polyethylene glycol method (Chiu et al. 1996; Chen et al. 2006). The corresponding amino acid sequence of the NLS protein was MDPKKKRKV. We observed the fluorescence of the samples above at an excitation wavelength of 588 nm and an emitting wavelength of 635 nm under a confocal laser-scanning microscope (Olympus FV1000). The primer sequences are listed in Supplementary Table S2.

### Quantitative RT-PCR analysis

Using the RNApure Plant Kit (Beijing CoWin Biotech Co. Ltd, Beijing, China), total RNA was extracted from roots, culms, leaf blades, leaf sheaths, young panicle from stage 3–8, and young pistils at stage 8 of inflorescence developmental course (Ikeda et al. 2004; Itoh et al 2005) sampled from Nipponbare and SSSL14. RNase-free DNase I was used to remove the genomic DNA in the sample. cDNA at a volume of 20 μl was synthesised using 1 μg RNA using the HiScript II 1st Strand cDNA synthesis Kit (Vazyme biotech co., Ltd, Nanjing, China). Quantitative RT-PCR (20 μl reaction volume) was carried out using 0.4 μl of cDNA, 0.4 μM of each gene-specific primer, and the AceQTM qPCR Kit (Vazyme) in the Roche Applied Science LightCycler™ 480 (Roche diagnostics Ltd., Germany). The ubiquitin gene was used as the internal controls. The primers used for qRT-PCR are listed in Supplementary Table S2. The corresponding PCR programme was pre-denaturation at 95 °C for 5 min, denaturation at 94 °C for 10 s, annealing at 60 °C for 30 s, and a total of 40 cycles. The relative expression of the target gene was calculated using the following formula: Exp = 2^−Δ*Ct*^, where Δ*Ct* = *Ct*_target gene_ − *Ct*_internal control_ (Livak and Schmittgen 2001).

### Pollen fertility observation

We stained the mature pollen grains of variety 9311, 7001S^*SYL3−n*^, and 7001S^*SYL3−k*^ using a 1% I_2_-KI water solution and examined them under a light microscope (Olympus BH-2) under 100× magnification.

### Phylogenetic tree construction

The amino acid sequences of the conserved parts of the MADS-box proteins in *Arabidopsis* and rice were obtained from the studies conducted by Alvarez-Buylla et al. (2000) and Parenicová et al. (2003) and the Plant Transcription Factor Database (http://plntfdb.bio.uni-potsdam.de/v3.0/). The amino acid sequences of the conserved parts of the MADS-box proteins in wheat and barley were obtained from http://www.ncbi.nlm.nih.gov/. Then, sequence alignment and construction of the neighbour-joining tree were performed using the MEGA5 software (Tamura et al. 2011) based on the maximum likelihood method.

### F_1_ hybrid seed production potential evaluation for the ***SYL3*** alleles in the paddy field

In order to evaluate the potential of the *SYL3* gene in the production of F_1_ hybrid rice seeds, we conducted an actual F_1_ seed production experiment using isogenic lines 7001S^*SYL3−n*^ (short SYL) and 7001S^*SYL3−k*^ (long SYL) as the female parents and variety 9311 as the common male parent in the paddy fields of Jiangpu Experimental station, Nanjing. 7001S^*SYL3−n*^ is a long day-sensitive male sterile line used in commercial F_1_ seed production in Eastern China, and 7001S^*SYL3−k*^ is an isogenic line obtained in this study. The male and female parents were grown in a ratio of 2:6:2, i.e. 4 lines of 9311 plants were planted around 6 lines of female plants. For each combination, 30 m^2^ of land was utilised for planting. Before artificial pollination at the flowering time, we observed the fertility of the pollen of 7001S^*SYL3−n*^ and 7001S^*SYL3−k*^ under a light microscope to ensure that the pollen of the female parents was sterile. We performed artificial supplementary pollination twice per day during pollen dispersal. Thirty days after artificial supplementary pollination, we harvested the seeds from the female plants individually. The potential of the *SYL3* allele for hybrid rice seed production was evaluated according to the weight of rice grains harvested from the male sterile plants per 1.5 m^2^ of area.

### Geographical distribution and genetic diversity analyses of the *qSYL3* alleles

In order to analyse the molecular variation in the *SYL3* alleles, we collected 136 rice accession resources and designed primers to amplify the *SYL3* coding regions of these varieties (Supplementary Table S1). The sequencing was completed at Nanjing TsingKe biological technology Co., Ltd (Nanjing, China). The genetic pedigree diagram of the *SYL3* alleles in cultivated and wild rice was developed using the median-joining model of NETWORK version 5.0 (Bandelt et al. 1999). After sequencing, the nucleotide diversity of the cultivated rice groups (*indica*, *japonica*, and *javanica*) and wild rice group in 20 genes around *SYL3* (Supplementary Table S3) was analysed using the DnaSP 5.0 software (Librado and Rozas 2009).

### Coalescent simulations

Coalescent simulation was used to model the bottleneck impact on sequence diversity using Hudson’s *ms* programme (Hudson 2002). We modelled the divergence of the cultivated rice (*O. sativa*) population and wild rice (*O. rufipogon* and *O. nivara*), with a population bottleneck in the cultivated rice. We used the bottleneck model reported by Zhu et al. (2007) in this study (Supplementary Figure S1). We combined *O*. *rufipogon* and *O*. *nivara* as a single population as the progenitor of *O. sativa* following the report by Londo et al. (2006). Based on the bottleneck model, we assumed that a single ancestral population of size *Na* experienced an instantaneous size shift to a bottlenecked population of size *Nb* at time *t2* generation ago and the bottleneck population expanded instantaneously to the present population of size *Np* at time *t1* generation ago. The parameter *d*, the duration of the bottleneck, and the parameter *Nb* were used for the bottleneck. The parameter *K*, which is the ratio of *Nb* and *d*, was used to describe the severity of the bottleneck in domestication (Wright and Gaut 2005).

The parameter *d* (200, 500, 1000, 1500, 2000, and 3000) was set as per the reports by Zhu et al. (2007) and Asano et al. (2011). We first set *Na* to be 120,000 to perform the simulation. A grid of 20 K values is listed in Supplementary Table S4, and the corresponding *Nb*, which is *K* × *d*, is also shown. We simulated the average number of SNP patterns in a 100-kb region corresponding with the region from genes 6–17 listed in Supplementary Table S3.

## Results

### Verification of *LOC_Os03g14850* as the gene *SYL3* using transgenic complementation and overexpression experiments

Within the 19.8-kb chromosome region harbouring the *qSYL3* locus, three annotated genes, namely *LOC_Os03g14850*, *LOC_Os03g14860*, and *LOC_Os03g14880,* were found according to the rice genome annotation database. Among these annotated genes, *LOC_Os03g14850* was determined as the candidate gene of *SYL3* according to the results of the qRT-PCR and T-DNA insertion (Liu et al. 2015b). *LOC_Os03g14850* is predicted to encode a MADS-domain family (MADS is named for yeast MCM1, plant AGAMOUS and DEFICIENS, and mammal Serum Response Factor) SRF-like protein. According to the results of the neighbour-joining tree, all the MADS-box proteins are divided into two groups (Type I and Type II) with more than 95% supports, and Type I is further divided into three subgroups (Mα, Mβ, and Mγ). *LOC_Os03g14850* is in the subgroup Mα in the Type I group and shares 78% amino acid identity with gene *LOC_Os06g22760* (Supplementary Figure S2A). As shown in Figure S2B, *LOC_Os03g14850* contains one M domain (8–57 aa) at its *N*-terminal region. We also found that the structure of *LOC_Os03g14850* is different from that of the other members in the MADS-box family because it contained the least number of exons (Supplementary Fig. S2C).

**Fig. 1:**
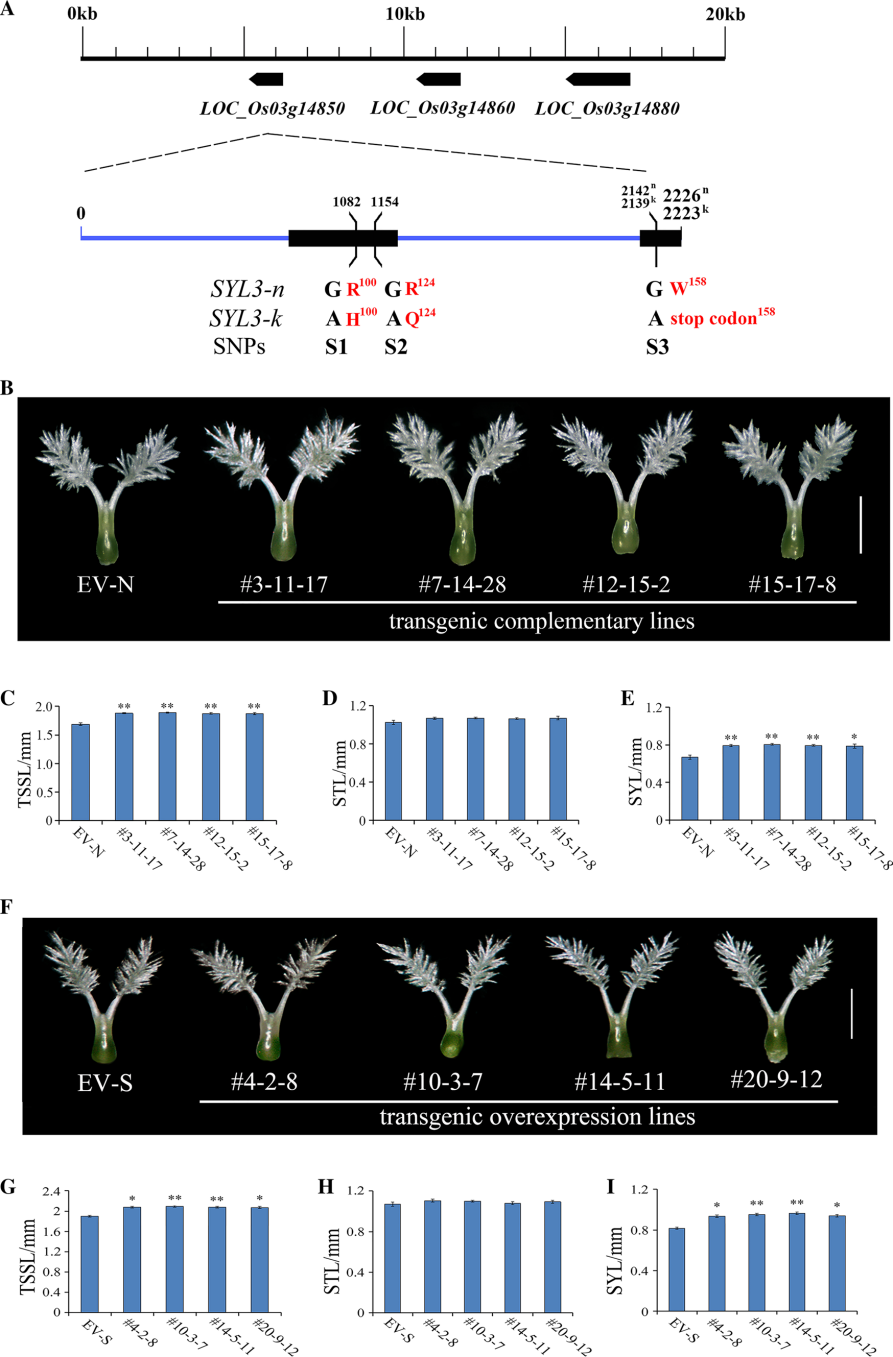
Gene structure of *SYL3* and the phenotypes identification of *SYL3* complementary and overexpression strains. **A** Gene structure of *SYL3* and natural variation between the Nipponbare (*SYL3-n*) and Kasalath (*SYL3-k*) alleles. The black boxes denote exons, and the blue line denotes introns. **B** Pistil morphology of four independent transgenic complementary lines and Nipponbare with empty vector (EV-N) as a control. Scale bar, 1 mm. **C**–**E** Comparison of the phenotype between transgenic complementary lines and EV-N. Data represent means ± SD (*n* = 43 independent plants). **F** Pistil morphology of four independent transgenic overexpression lines and SSSL14 with empty vector (EV-S) as a control. Scale bar, 1 mm. **G**–**I** Comparison of the phenotype between transgenic overexpression lines and EV-S. Data represent means ± SD (*n* = 52 independent plants). *P < 0.05, **P < 0.01, Student’s *t*-test. TSSL, the sum of stigma and style length; STL, stigma length; SYL, style length

Three SNPs were identified in the CDS of *LOC_Os03g14850* between the Nipponbare allele (designated *SYL3-n*) and Kasalath allele (designated *SYL3-k*) (Fig. 1A). A 4223-bp genomic fragment from Kasalath was able to fully increase TSSL and SYL and had no effect on the STL in the transgenic complementation lines comparing with the Nipponbare plants with the empty vector (EV-N) (Fig. 1B–E). Furthermore, the transgenic overexpression plants with increased expression of *SYL3-k* showed longer TSSL and SYL and no change in the STL compared with the SSSL14 plants with the empty vector (EV-S) (Fig. 1F–I). Thus, we concluded that *LOC_Os03g14850* was the *SYL3* gene.

**Fig. 2:**
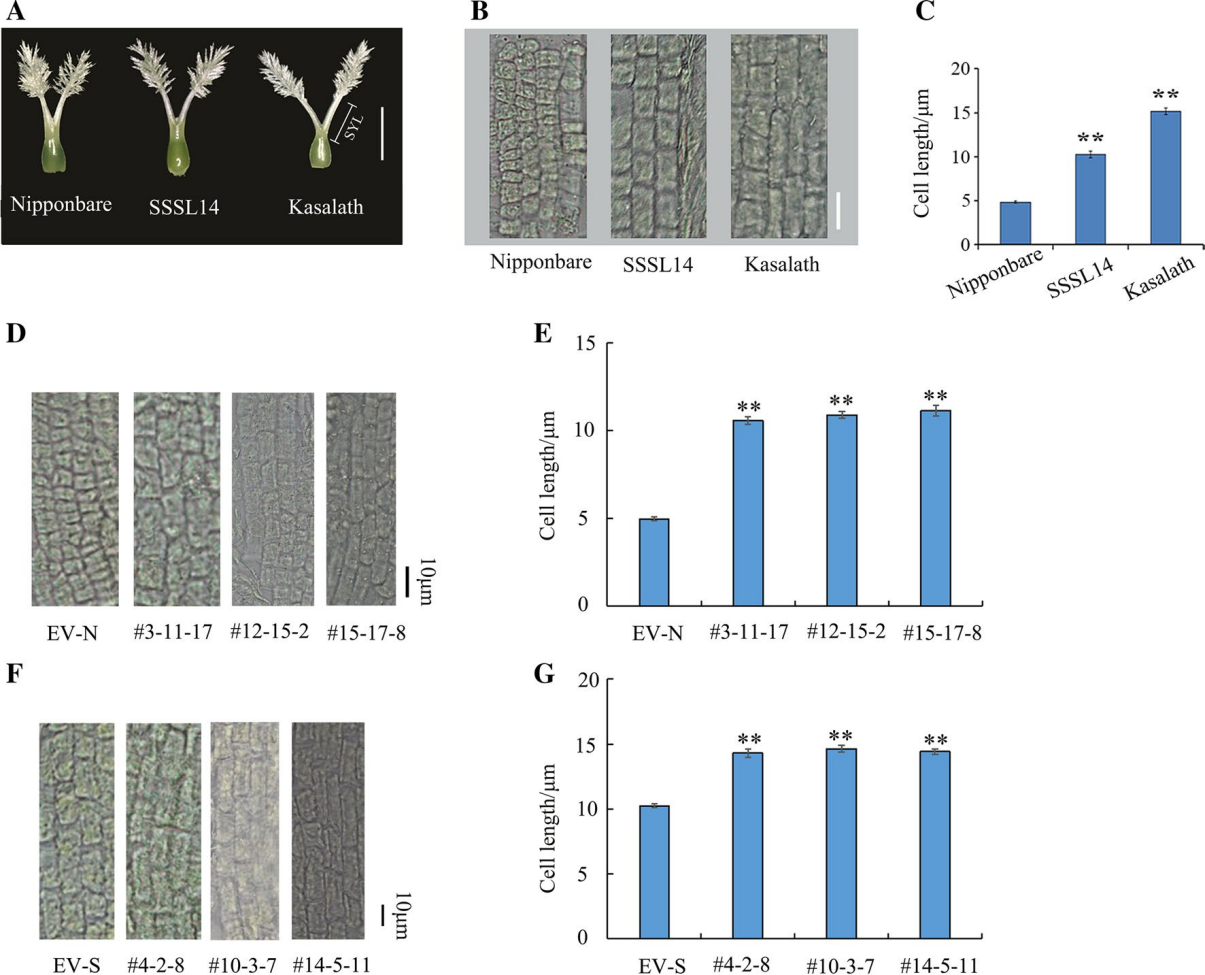
Morphology display of pistil and cell in style part for Nipponbare, SSSL14 and Kasalath. **A** Pistil morphology viewed by stereomicroscopy. Scale bar, 1 mm. **B** Cell morphology of style. Scale bar, 10 μm. **C** Comparison of cell length among Nipponbare, SSSL14 and Kasalath. Data represent means ± SD (*n* = 40 independent plants). **D** Cell morphology for EV-N and transgenic complementary line #3-11-17, #12-15-2 and #15-17-8. Scale bar, 10 μm. **E** Comparison of cell length between EV-N and #3-11-17, #12-15-2 and #15-17-8. Data represent means ± SD (*n* = 24 independent plants). **F** Cell morphology for EV-S and #4-2-8, #10-3-7 and #14-5-11. Scale bar, 10 μm. **G** Comparison of cell length between EV-S and #4-2-8. Data represent means ± SD (*n* = 30 independent plants). **P < 0.01, Student’s *t*-test

Figure 2A shows the pistil morphology of Nipponbare, SSSL14, and Kasalath. As reported by Liu et al. (2015a, b), there were no significant differences in the STL among Nipponbare, SSSL14, and Kasalath. The SYL of Nipponbare was significantly shorter than that of SSSL14 and Kasalath (Table 1). The cell length of the style in Nipponbare (3.53 ± 0.61 μm) was significantly shorter than that of SSSL14 (9.67 ± 1.56 μm) and Kasalath (12.63 ± 1.95 μm) (*P* < 0.01) (Table 1; Fig. 2B, C). We also found that the cell length in the style of the complementation and overexpression lines was significantly longer than that of their respective controls (Fig. 2D–G). These results indicated that the *SYL3-k* allele increased SYL via the elongation of cell length in style.

**Table 1:** Comparisons for TSSL, STL, SYL of pistil and cell length of style in Nipponbare, SSSL14 and Kasalath, respectively

Materials	Mean ± standard deviation for traits			
	TSSL/mm	STL/mm	SYL/mm	Cell length in style/μm
Nipponbare	1.73 ± 0.10	1.03 ± 0.11	0.71 ± 0.06	3.53 ± 0.61
SSSL14	1.93 ± 0.08**	1.07 ± 0.08	0.85 ± 0.05**	9.67 ± 1.56**
Kasalath	2.13 ± 0.11**	1.11 ± 0.11	1.02 ± 0.08**	12.63 ± 1.95**

*TSSL* the sum of stigma and style length, *STL* stigma length, *SYL* style length

**Indicates significant difference at *α* = 0.01 probability level compared with Nipponbare. Student’s *t*-test

In addition, significant differences were observed between the EV-N plants and complementation lines in the plant height (*P* < 0.01) (Supplementary Fig. S3A), panicle exertion length (*P* < 0.05) (Supplementary Fig. S3B), and panicle length (*P* < 0.01) (Supplementary Fig. S3C). We also observed significant differences between the EV-S plants and overexpression lines in terms of plant height (*P* < 0.01) (Supplementary Fig. S3D), panicle exertion length (*P* < 0.05) (Supplementary Fig. S3E), and panicle length (*P* < 0.05) (Supplementary Fig. S3F). From these results, we speculated that *LOC_Os03g14850* had a pleiotropic effect to control plant height, panicle length, and panicle exertion length.

### *qSYL3* is constitutively expressed gene and expresses in nuclear

**Fig. 3:**
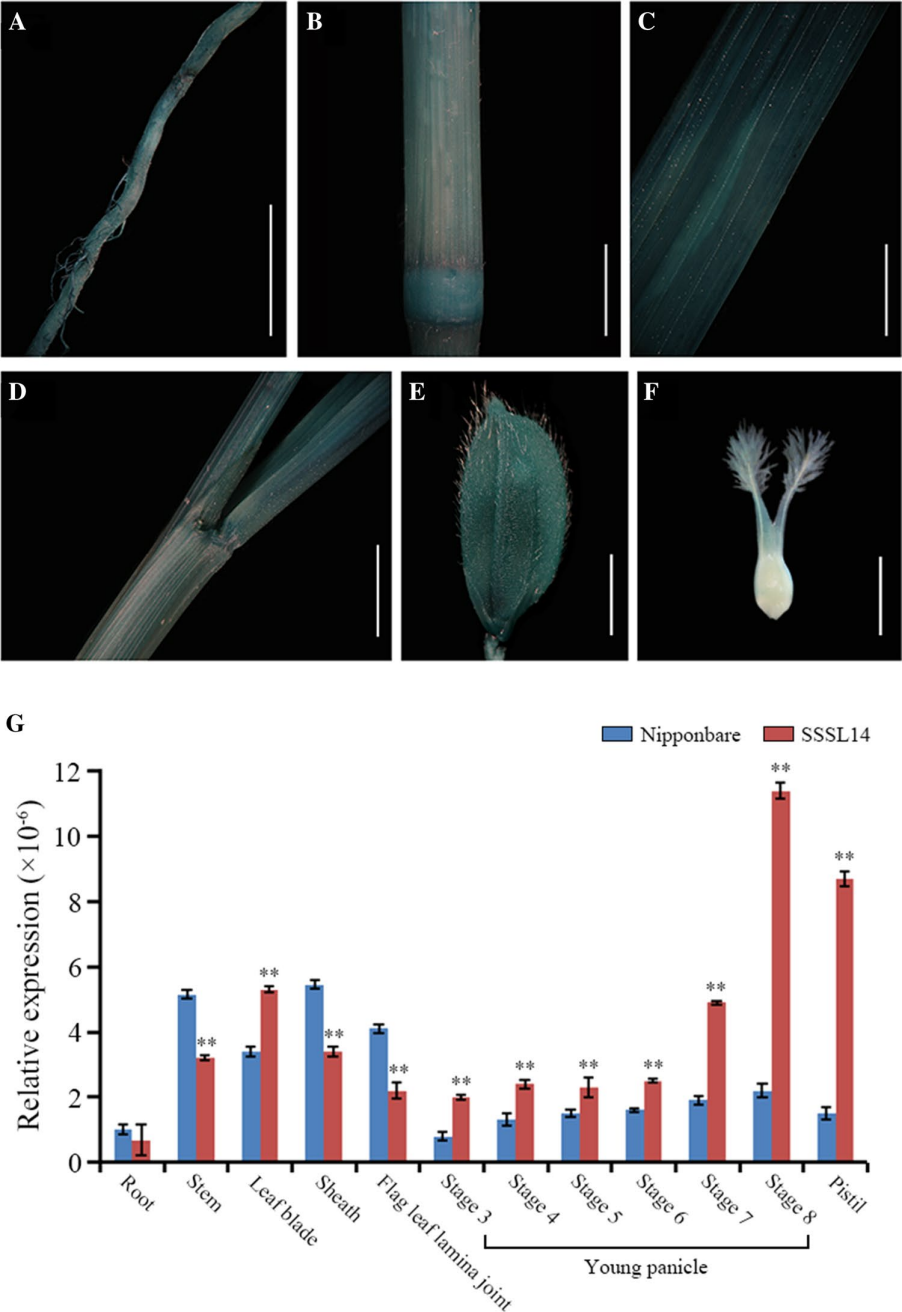
Expression pattern of *SYL3*. **A**–**F** The GUS expression pattern in the *SYL3-k:GUS* transgenic plant. **A** Root, Scale bar, 2 cm; **B** stem, Scale bar, 1 cm; **C** leaf blade, scale bar, 1 cm; **D** leaf sheath, scale bar, 1 cm; **E** spikelet, Scale bar, 1 mm; **F** pistil, Scale bar, 1 mm. The experiment was repeated 3 times independently with similar results. **G** Tissue-specific expression pattern of *SYL3-k* revealed by qRT-PCR. The ubiquitin gene was used as an internal control. Data represent means ± SD. (*n* = 3 biologically independent samples). *P < 0.05, **P < 0.01, Student’s *t*-test

To examine the spatiotemporal expression patterns of *SYL3-k*, we detected GUS signals in the root, culm, leaf blade, leaf sheath, and pistil of the p*SYL3-k*::GUS transgenic plants of Nipponbare and found that all the examined tissues showed a significant GUS signal (Fig. 3A–F). Furthermore, we performed qRT-PCR analysis of the endogenous *SYL3* transcripts using the total RNA isolated from different tissues and same tissues at different development stages of plants Nippponbare and SSSL14, respectively. As shown in Fig. 3G, *SYL3* transcripts were detected in all the tissues examined, but its expression was most abundant in the young panicle and pistil at stage 8 of inflorescence differentiation in Nippponbare and SSSL14. SSSL14 (*SYL3-k*) is more highly expressed in pistil tissues compared to Nippponbare (*SYL3-n*). These results indicated that *SYL3* is a constitutively expressed gene.

To determine the subcellular localisation of SYL3, we fused the full-length CDS of *SYL3-k* with that of RFP. The nuclear localisation signal and mCherry fusion protein were used as the nuclear marker. The transient expression experiments in rice leaf protoplasts showed that RFP-SYL3 was specifically localised in the nucleus (Supplementary Fig. S4), providing evidence that SYL3 expresses in nuclear.

### The effect of ***SYL3-k*** on style elongation is associated with higher GA_4_ levels in pistil, which might be caused by higher expression level of ***OsGA3ox2***

We examined GA_1,_ GA_4_, BR, and IAA levels in the pistil tissues of Nipponbare and SSSL14 to explore the cause of cell length elongation in the style. The results showed that the GA_4_ content in the pistils of the Nipponbare plants was 7.01 ng/g, which was significantly lower than that in SSSL14 plants (9.19 ng/g) (Table 2). There were no significant differences in the GA_1_ content in the pistils between the Nipponbare and SSSL14 plants (Table 2). There was no BR detected in the pistils of both the Nipponbare and SSSL14 plants. The IAA content in the pistils of Nipponbare was not significantly different from that of SSSL14 (Table 2). Thus, we concluded that the cell length elongation in the style tissues resulted from the increase in the GA_4_ content in the pistil.

**Table 2:** Detection result of endogenous hormone GA_4_, GA_1,_ BR and IAA for Nipponbare and SSSL14

Hormone	Sample name	Average detection concentration^a^/ng/ml	Mass coefficient/g	Volume coefficient/ml	Hormone content ng/g
GA_4_	Nipponbare	3.05	0.087	0.200	7.01
	SSSL14	3.54	0.077	0.200	9.19**
GA_1_	Nipponbare	0.80	0.531	0.200	0.30
	SSSL14	1.10	0.591	0.200	0.37
BR	Nipponbare	0	0.519	0.200	0
	SSSL14	0	0.732	0.200	0
IAA	Nipponbare	0.80	0.433	0.400	0.74
	SSSL14	1.12	0.651	0.400	0.69

**Indicates significant at *α* = 0.01 probability level

^a^Indicates the average value of three repeats

**Fig. 4:**
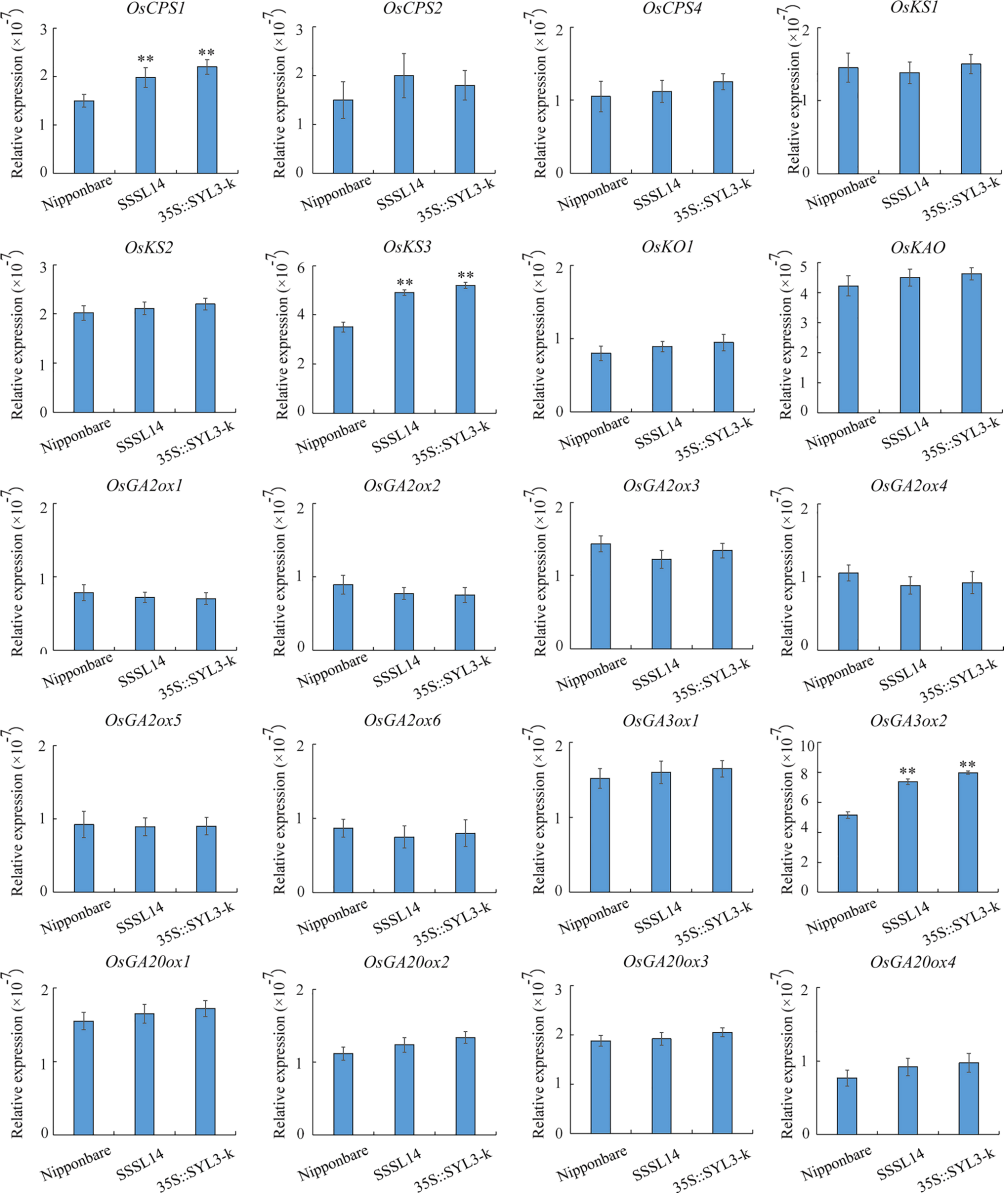
qRT-PCR analysis of genes associated with GA synthesis pathway in Nipponbare, SSSL14 and transgenic 35S::SYL3-k overexpression line. The relative expression level of each gene in pistil at stage 8 of young panicle differentiation of Nipponbare, SSSL14 and transgenic 35S::SYL3-k overexpression line were analysed by qRT-PCR and normalised using the ubiquitin gene as an internal control. Data are means ± SD (*n* = 3). Asterisks indicate statistically significant differences compared with the wild type at *P* < 0.01 by Student’s *t* test

To understand the mechanism of action of *SYL3* in the regulation of GA_4_ content, the expression patterns of 20 key genes in the gibberellin (GA) biosynthesis pathway in rice pistils at stage 8 of young panicle differentiation were investigated using qRT-PCR. When the ubiquitin gene was used as the internal control, the qRT-PCR results showed that the expression level of *OsCPS1*, *OsKS3,* and *OsGA3ox2* in SSSL14 and transgenic 35S::SYL3-k overexpression line was significantly higher than those in Nipponbare (Fig. 4), but the expression level of the other 17 genes showed no significant differences. Considering the study that demonstrated that the expression level of *OsCPS1* and *OsKS3* did not affect the content of bioactive GAs (Sakamoto et al. 2004), we speculated that the higher expression level of *OsGA3ox2* in the plants with *SYL3-k* allele is likely to be related to the increasing content of GA_4_ in the SSSL14 plants.

**Fig. 5:**
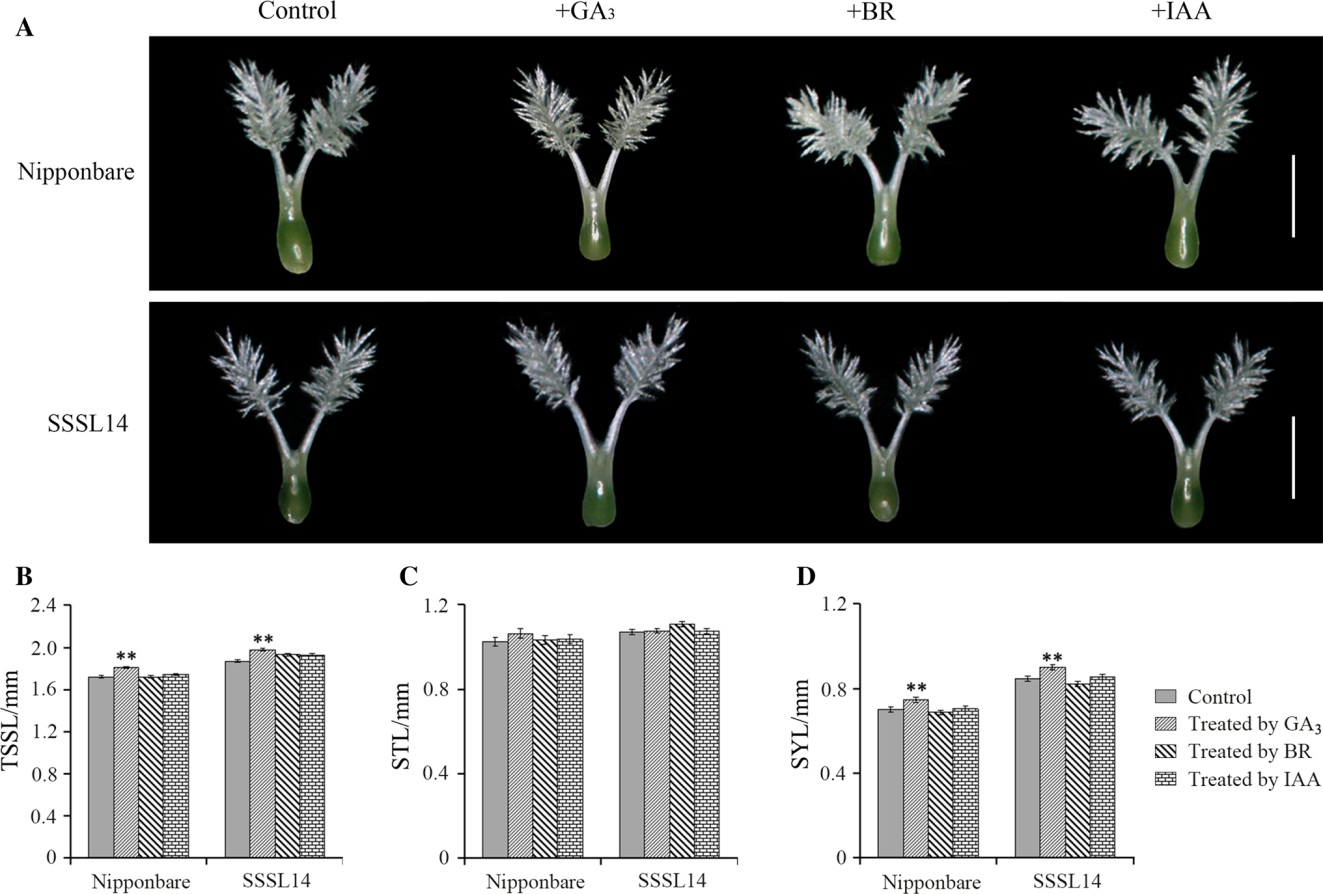
Pistil morphological display and length comparison between control and treatment of GA_3_, BR and IAA in Nipponbare and SSSL14

To determine whether *SYL3-k* affects GA biosynthesis or signal transduction, we applied exogenous hormone GA_3_ solution to smear the flag leaf blade surface at stage 5 of inflorescence differentiation in Nipponbare and SSSL14 plants. The results showed that there were significant differences in the SYL, but not in the STL between the plants treated with water and those treated with GA_3_ in Nipponbare (Fig. 5). Additionally, no significant differences were found in the SYL and STL between the plants treated with water, and those treated with BR or IAA in Nipponbare (Fig. 5). Similar results were obtained in the SSSL14 plants (Fig. 5). These results suggested that *SYL3* might be involved in GA biosynthesis, but not in signal transduction.

To further explore whether *SYL3* can be involved in GA signal transduction, four key genes for GA signal transduction were selected for qRT-PCR analysis. Previous studies revealed that genes *GID1*, *GID2*, *OsSPY*, and *SLR1* up-regulated GA signal transduction, but there were no significant expression level differences among Nipponbare, SSSL14 and transgenic 35S::SYL3-k overexpression line (Supplementary Figure S5). These results indicated that *SYL3-k* was not involved in GA signal transduction mediated by genes *GID1*, *GID2*, *OsSPY*, and *SLR1*.

### Yield of the F_1_ hybrid seeds harvested from the male sterile line with the ***SYL3-k ***allele was significantly higher than that with the ***SYL3-n*** allele

**Fig. 6:**
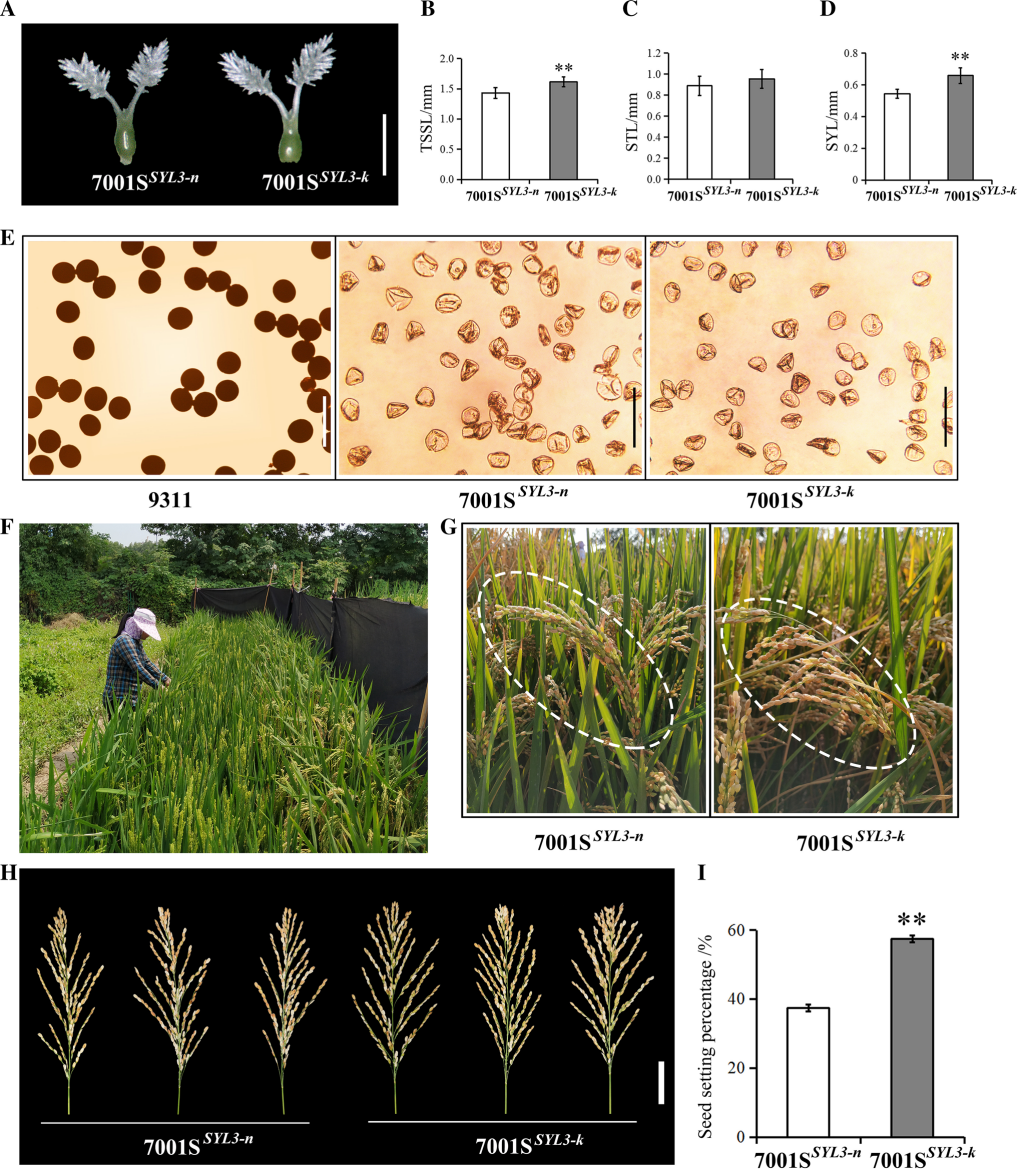
Hybrid rice seed production evaluation of *SYL3* gene. **A** Pistil morphology of 7001S^*SYL3−n*^ and 7001S^*SYL3−k*^. Scale bar, 1 mm. **B** Comparison of the TSSL of 7001S^*SYL3−n*^ and 7001S^*SYL3−k*^. **C** Comparison of the STL of 7001S^*SYL3−n*^ and 7001S^*SYL3−k*^. **D** Comparison of the SYL of 7001S^*SYL3−n*^ and 7001S^*SYL3−k*^. Data represent means ± SD (*n* = 40 independent plants), **P < 0.01, Student’s *t*-test. **E** Pollen viability test. Fertile pollen grains of 9311 could be stained by 1% I_2_-KI, while abortive pollen grains of 7001S^*SYL3−n*^ and 7001S^*SYL3−k*^ could not be stained. Scale bar, 100 μm. **F** Operation of artificial supplementary pollination with a bamboo pole. **G** Field performance of 7001S^*SYL3−n*^ and 7001S^*SYL3−k*^. **H** Panicle morphology of 7001S^*SYL3−n*^ and 7001S^*SYL3−k*^. Scale bar, 5 cm. **I** Comparison of the seed setting percentage of 7001S^*SYL3−n*^ and 7001S^*SYL3−k*^. Data represent means ± SD (*n* = 30 independent plants), **P < 0.01, Student’s *t*-test

The SYL of 7001S^*SYL3−k*^ was significantly longer than that of 7001S^*SYL3−n*^ (Fig. 6A–D). To further evaluate the potential of the *SYL3-k* allele in hybrid rice seed production, we performed a field experiment using the two combinations, 7001S^*SYL3−n*^ × 9311 and 7001S^*SYL3−k*^ × 9311. The pollen of plants in 7001S^*SYL3−n*^ and 7001S^*SYL3−k*^ could not be stained by a 1% solution of I_2_-KI and was completely sterile (Fig. 6E). Therefore, the F_1_ seeds were obtained by artificial supplementary pollination at the flowering stage (Fig. 6F). The stigma exertion rate of 7001S^*SYL3−k*^ was 25.8%, which is significantly higher than that of 7001S^*SYL3−n*^ (16.3%). The outcrossing seed setting rate of the 7001S^*SYL3−k*^ × 9311 combination was 57.4%, which is significantly (*P* < 0.05) higher than that of the 7001S^*SYL3−n*^ × 9311 combination (37.2%) (Fig. 6G–I). The weight of the F_1_ seeds harvested from the female parents in the 1.5 m^2^ area for the combination of 7001S^*SYL3−k*^ × 9311 was 581.07 g, which is significantly (*P* < 0.05) higher than that of the combination of 7001S^*SYL3−n*^ × 9311 (501.98 g) in the same area of land. These results indicate that the *SYL3-k* allele could significantly (*P* < 0.05) increase the yield of the F_1_ hybrid seeds via enhancing the outcrossing rate of the male sterile lines.

### Identification of functional nucleotide polymorphisms (FNPs) in *SYL3*

To explore the molecular basis of the functional differences between the *SYL3-n* and *SYL3-k* alleles in regulating SYL, the CDS of *SYL3-n* and *SYL3-k* were compared. Following the comparisons, three SNP sites at positions 1082 (S1: G/A), 1154 (S2: G/A), and 2142 (S3: G/A) were found (Fig. 1A). For S1, the base G in the *SYL3-n* allele was changed to base A in the *SYL3-k* allele, leading to a change of the amino acid from Arg to His (R100H). For S2, the base G in the *SYL3-n* allele was changed to base A in the *SYL3-k* allele, leading to a change of the amino acid from Arg to Gln (R124Q). For S3, the base G in the *SYL3-n* allele was changed to base A in *SYL3-k* allele, leading to a change of the amino acid from Trp to the stop codon (W158 Stop codon) (Fig. 1A). We then compared the *SYL3* coding region sequences of 101 cultivated rice accessions and did not find additional polymorphisms (Supplementary Table S1). The simple association between the three SNPs and the pistil traits of the 101 cultivated rice accession resources was analysed. Our results indicate that SNP positions S1 and S2 are both significantly associated with the SYL (Supplementary Table S5).

To verify the effects of S1 and S2, we identified the pistil phenotypes of the transgenic plants of four haplotypes, namely H1, H2, H3, and H4 (Supplementary Fig. S6A). The H1 (S1A/S2A) and H4 (S1G/S2G) constructs are identical to SSSL14 and Nipponbare, respectively. H2 is the S1G/S2A recombinant CDS, whereas H3 is the S1A/S2G recombinant CDS (Supplementary Figure S6A). We found that the transgenic positive plants and the transgenic negative plants followed the segregation ratio of 3:1 by detecting the T_2_ generation transgenic plant population. Except for the transgenic plants of H4, the transgenic positive plants of H1, H2, and H3 had an increase in SYL, among which the transgenic positive plants of H1 demonstrated the strongest effect (Supplementary Figure S6B–F). In conclusion, S1 and S2 are the FNPs that regulate the SYL.

### Geographic distribution and genetic diversity analyses of the *SYL3* alleles

To examine the evolutionary origin of the *SYL3* alleles, we performed a network analysis based on the sequences of *SYL3* coding regions in 35 wild rice accessions and 101 cultivated rice accessions (Supplementary Fig. S6G). The wild rice accessions, which were distributed at latitudes S16°–N28°, included the H1, H2, and H3 haplotypes (Supplementary Table S1). The *indica* rice accessions, which were distributed at N5°–N38°, included the H1, H2, H3, and H4 haplotypes (Supplementary Table S1). The *javanica* rice accessions, which were distributed at S12°–N21°, included the H3 and H4 haplotypes (Supplementary Table S1). The *japonica* rice accessions, which were distributed at N22°–N45°, included the H4 haplotype (Supplementary Table S1). During the domestication of rice from the south to north, haplotypes H1, H2, and H3 gradually disappeared after the artificial selections and the haplotype H4 remained. The H4 haplotype plays an important role for the northward expansion of *japonica* to regions with naturally long days in Asia.

We examined the four haplotypes composed of S1 and S2 sites of functional SNPs in the CDS sequence of *SYL3* gene locus of 108 male sterile lines. It was found that 21 *japonica* male sterile lines (16 of which were cytoplasmic nuclear interaction male sterile lines and 5 were photosensitive male sterile lines) were all H4 haplotypes, while among 87 *indica* male sterile lines (45 of which were cytoplasmic nuclear interaction male sterile lines and 42 were photo-thermo-sensitive male sterile lines), three haplotypes, i.e. H2, H3, and H4, were detected (Supplementary Table S6). This result is similar to the identification result of the natural population composed of 101 cultivated varieties. In fact, the cytoplasmic nuclear interaction male sterile line is produced by replacing the normal fertile cytoplasm in the maintainer line with the sterile cytoplasm; in contrast, photo-thermo-sensitive genic male sterile line is produced by introducing photo-thermo-sensitive genic male sterile gene into the nuclear genome of normal fertile varieties. Therefore, the nuclear genome of maintainer lines (or excellent varieties to be transformed into photo-thermo-sensitive male sterile lines) basically determines the nuclear genome of male sterile lines. Because maintainer lines (or excellent varieties) were selected from the population of bred varieties, the corresponding alleles at *SYL3* locus are inevitably artificially selected.

To test the genetic differences between *indica* and *japonica* subpopulations, we sequenced and calculated the nucleotide diversity for 20 genes around the *SYL3* locus. The results showed that the average nucleotide diversity of the 20 genes for *japonica* (*π*_japonica_ = 0.017) was lower than that for *indica* rice (*π*_indica_ = 0.069), *javanica* rice (*π*_javanica_ = 0.036), and wild rice (*π*_wild rice_ = 0.141). We also investigated the nucleotide diversity values (*π*) and *F*_*ST*_ ratio (*japonica* rice accessions/*indica* rice accessions) values in the *SYL3* coding sequence, flanking region (surrounding 40-kb, referring to Gao et al. 2019; Yang et al. 2019; Gao et al. 2020), and the whole genome using a diversity panel of the 3 K rice genome project. The results show that the π values of the *SYL3* locus CDS in *japonica* rice accessions were lower than those in *indica* rice accessions and wild rice rufipogon accessions and were also lower than those in its flanking regions (Supplementary Fig. S7A). Meanwhile, the *F*_*ST*_ value in *SYL3* was obviously above the genome-wide threshold 0.087 (Supplementary Fig. S7B), suggesting the genetic difference in *SYL3* locus and its flanking regions between *indica* and *japonica* subpopulations.

In order to further elucidate that the low genetic diversity of *japonica* rice is caused by artificial selection rather than a domestication bottleneck effect, a coalescent simulation was performed with two derived populations (wild rice and cultivated rice). After the simulation, the *K* value for the 20 genes around *SYL3* was 0.01, which is much lower than the *K* value (0.2) reported by Zhu et al. (2007). These results suggested that the low gene diversity surrounding *SYL3* could not be simply explained by the domestication bottleneck effect. The selective sweep around the gene *SYL3* provides additional crucial evidence that *SYL3* may experience artificial selection during the domestication or improvement in *japonica* rice.

## Discussion

In this study, we found that there were no significant differences in the STL among Nipponbare, SSSL14, and Kasalath. The SYL of SSSL14 and Kasalath was significantly longer than that of Nipponbare (Table 1). The finding that the SYL, rather than the STL, varies with the latitude has an important significance in biology and agriculture. The main reasons for this are as follows: First, the stigma is an organ receiving/capturing pollen and is critical for seed setting and reproduction. The STL does not change easily. The longer the STL, the better it is at capturing pollen. Second, the style is merely a passage that allows the pollen tube to reach the ovary. The SYL can be long or short. However, a short SYL can save the energy of the plant, which is beneficial. The shorter the SYL, the better chances of the pollen tube reaching the ovary. Therefore, the change in TSSL is mainly caused by the change of the SYL, which is kind of an environmental adaptation of rice cultivation towards the Northern Hemisphere, and is a new discovery brought to light by this study.

Previous studies indicated that endogenous phytohormones, such as GAs, BR, and auxin IAA, play many important roles throughout plant development like stem and cell elongation and pollen tube growth (Kobayashi et al. 1988; Clouse and Sasse 1998; Steber and McCourt 2001; Ozga and Reinecke 2003; Martinelli et al. 2009; Pomares-Viciana et al. 2017). According to the reports by Kobayashi et al. (1994) and Yamaguchi (2008), we know that GA_1_ and GA_4_ are the major bioactive GAs in regulating the growth of rice, there is no endogenous GA_3_ in rice plants (Kobayashi et al. 1994), and GA_3_ and GA_7_ mainly exist in the fungus *Gibberella fujikuroi* (Tudzynski et al. 2003; Yamaguchi 2008). Therefore, we examined GA_1,_ GA_4_, BR, and IAA levels in the pistil tissues of Nipponbare and SSSL14 to explore the cause of cell length elongation in the style. We found that the change in the SYL is caused by a change in the GA_4_ content in pistils, which is another novel discovery made in this study. We also found that the increase in GA_4_ content could elongate the cell length in the style to increase the SYL, and thus TSSL, which was beneficial in increasing the exertion rate of the stigma and increasing the yield of hybrid rice seed production.

Based on the endogenous phytohormone detection, we found that the content of GA_4_ in SSSL14 was significantly higher than that in Nipponbare (Table 2). Through the exogenous GA_3_ smears, it was found that SYL of Nipponbare and SSSL14 could be significantly elongated (Fig. 5). These results suggested that *SYL3-k* was related to GA biosynthesis, but not related to the GA signal transduction. Furthermore, we investigated the expression patterns of 20 key genes in the GA biosynthesis pathway in rice pistils and found that the expression levels of *OsCPS1*, *OsKS3,* and *OsGA3ox2* in SSSL14 and 35S::SYL3-k were significantly higher than those in Nipponbare. Sakamoto et al. (2004) reported that the expression level of *OsCPS1* and *OsKS3* did not affect the content of bioactive GAs. It is reported that BR greatly induces the expression of *OsGA3ox2*, one of the GA biosynthesis genes, leading to increased GA_1_ levels in rice seedlings (Tong et al. 2014). The growth of reproductive organs is regulated mainly by GA_4,_ and the vegetative growth is regulated by GA_1_ (Kobayashi et al. 1988, 1989). However, in this study, we did not detect BR in rice pistils. Therefore, we speculated that the higher expression of *OsGA3ox2* in the *SYL3-k* isogenic line was more likely to be related to increasing the GA_4_ content.

In this study, we also found a significant difference in *SYL3* locus and its flanking regions between *indica* and *japonica* subpopulation. First, based on the correlation analysis between the alleles and traits and site-directed mutation tests, we demonstrated that the two SNPs, S1 (1082:G/A) and S2 (1154:G/A), were the FNPs, which have four haplotypes (H1, H2, H3, and H4). Out of the four haplotypes, three haplotypes (H1, H2, and H3) had strong effects on the SYL and mainly existed in wild rice, and *indica* and *javanica* rice varieties, which grow in low latitude areas (Supplementary Fig. S6G). However, haplotype H4 had no effects on the SYL and mainly existed in *japonica*, which is planted in high latitude areas of the Northern Hemisphere (Supplementary Fig. S6G). Secondly, the nucleotide diversity (*π*) values of the *SYL3* locus coding sequence in japonica rice accessions are lower than those in indica rice accessions and wild rice rufipogon accessions and also lower than those in its the flanking regions (Supplementary Fig. S7A). Meanwhile, the *F*_*ST*_ value in *SYL3* was obviously above the genome-wide threshold 0.087 (Supplementary Fig. S7B).

In conclusion, our work revealed that the variation in SYL was caused by the variation in GA_4_ level in pistil, which is closely related to the variation in the expression level of *OsGA3ox2*. These results greatly improve our understanding of the causes of pistil length variation and provide the basis for the breeding of male sterile lines with high stigma exertion rate to increase the yield of F_1_ seed production.

## Supplementary Information

Below is the link to the electronic supplementary material.

Supplementary file1 (PDF 2382 KB)

Supplementary file2 (DOCX 26 KB)

Supplementary file3 (DOCX 14 KB)

Supplementary file4 (DOCX 14 KB)

Supplementary file5 (DOCX 15 KB)

Supplementary file6 (DOCX 14 KB)

Supplementary file7 (DOCX 16 KB)

## Data Availability

Data related to this manuscript are available within this paper and its Supplementary data.
