# Supplementary material for: SYL3-k increases style length and yield of F1 seeds via enhancement of endogenous GA4 content in Oryza sativa L. pistils
Source: Theor Appl Genet. 2021 Oct 17;135(1):321–36. doi: 10.1007/s00122-021-03968-y (PMC8741667; doi:10.1007/s00122-021-03968-y)
Supplement: Supplementary file 1 — Supplementary file1 (PDF 2382 KB) [file 122_2021_3968_MOESM1_ESM.pdf]

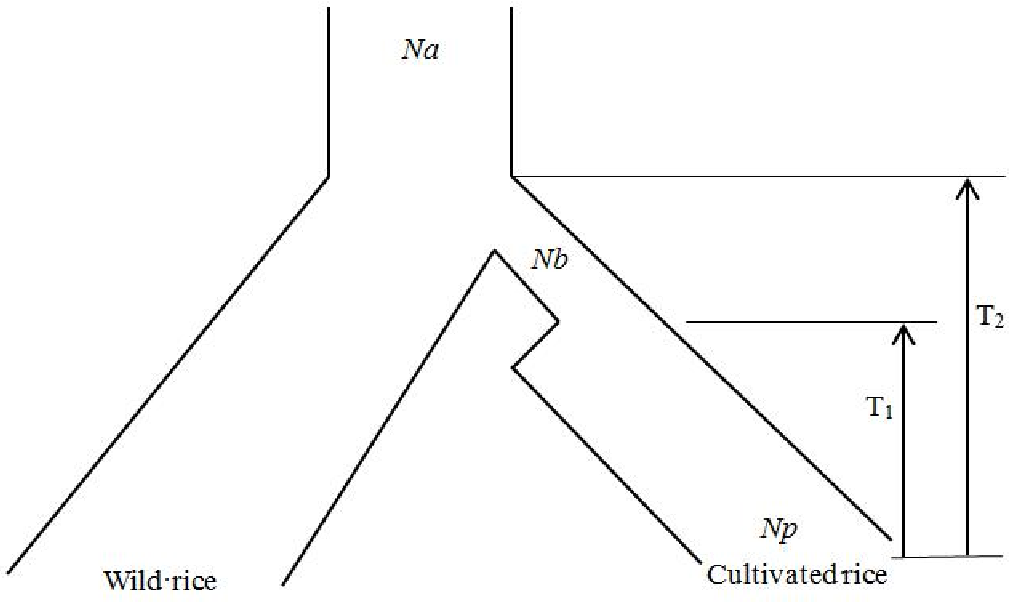

Figure S1 The single bottleneck model used in simulation. *Na*, the effective size of ancestor population; *Nb*, the effective size of cultivated rice during the bottleneck; *Np*, the effective size of cultivated rice; T_1, end time of the bottleneck; T_2, start time of the bottleneck; T_2-T_1, the duration of the bottleneck.

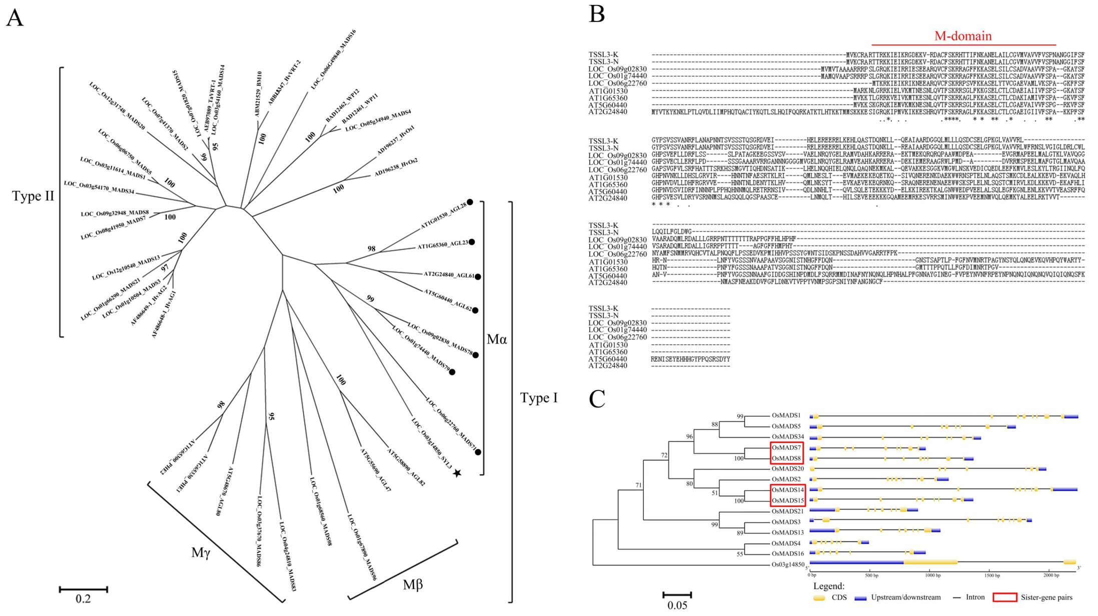

Figure S2 Neighbor-joining tree of MADS-box proteins, amino acid sequences alignment and gene structures of part *MADS-box* genes in rice. (A) Neighbor-joining tree of MADS-box proteins in *Arabidopsis*, rice, wheat and barley with bootstrap values above 95%. *SYL3* is indicated by an asterisk. The proteins used for the amino acid alignment are indicated by black circles. (B) Amino acid sequences alignment of *SYL3-n* (the Nipponbare allele), *SYL3-k* (the Kasalath allele), *MADS71* (*LOC_Os06g22760*, the closest homologue of *TSSL3* in the rice genome), *MADS79* (*LOC_Os01g74440*), *MADS78* (*LOC_Os09g02830*), *AGL62* (*AT5G60440*), *AGL61* (*AT2G24840*), *AGL23* (*AT1G65360*) and *AGL28* (*AT1G01530*). **(**C**)** Phylogenetic tree and structures of part *MADS-box* genes in rice. On the left is phylogenetic tree of *MADS-box* genes. On the right is the exon-intron structure of the *MADS-box* genes.

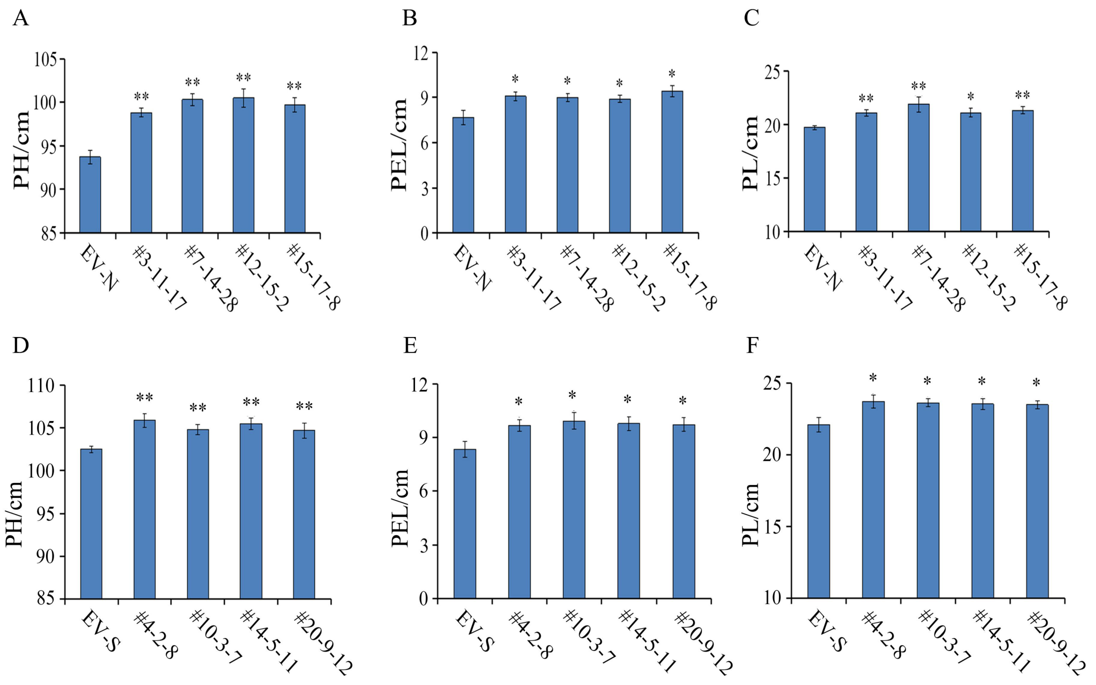

Figure S3 Comparison of plant height, panicle exsertion length and panicle length between transgenic lines and empty vector plants. (A-C) Comparison of PH (A), PEL (B), PL (C) between transgenic complementary lines and EV-N. (D-F) Comparison of PH (D), PEL (E), PL (F) between transgenic overexpression lines and EV-S. * $P<0.05$, ** $P<0.01$, Student's *t*-test. PH, plant height; PEL, panicle exsertion length; PL, panicle length.

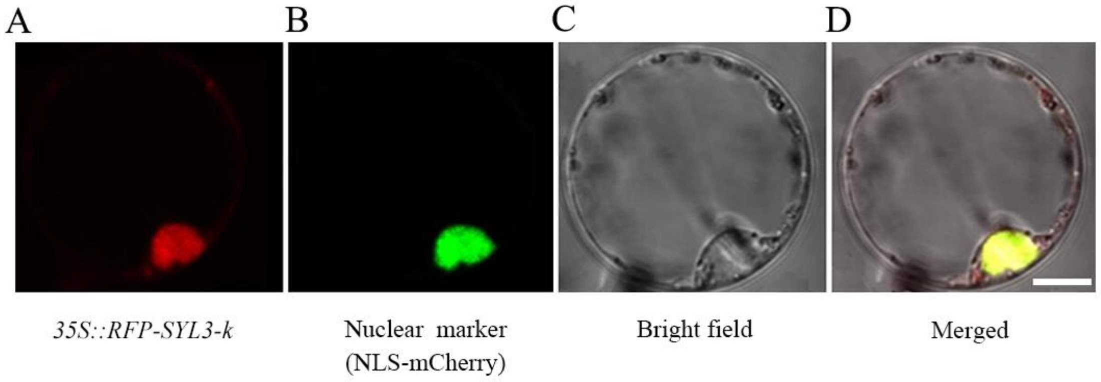

Figure S4 Subcellular localization detection of *SYL3*. The RFP-SYL3 fusion protein was exclusively expressed in the nucleus. Scale bar, 10μm.

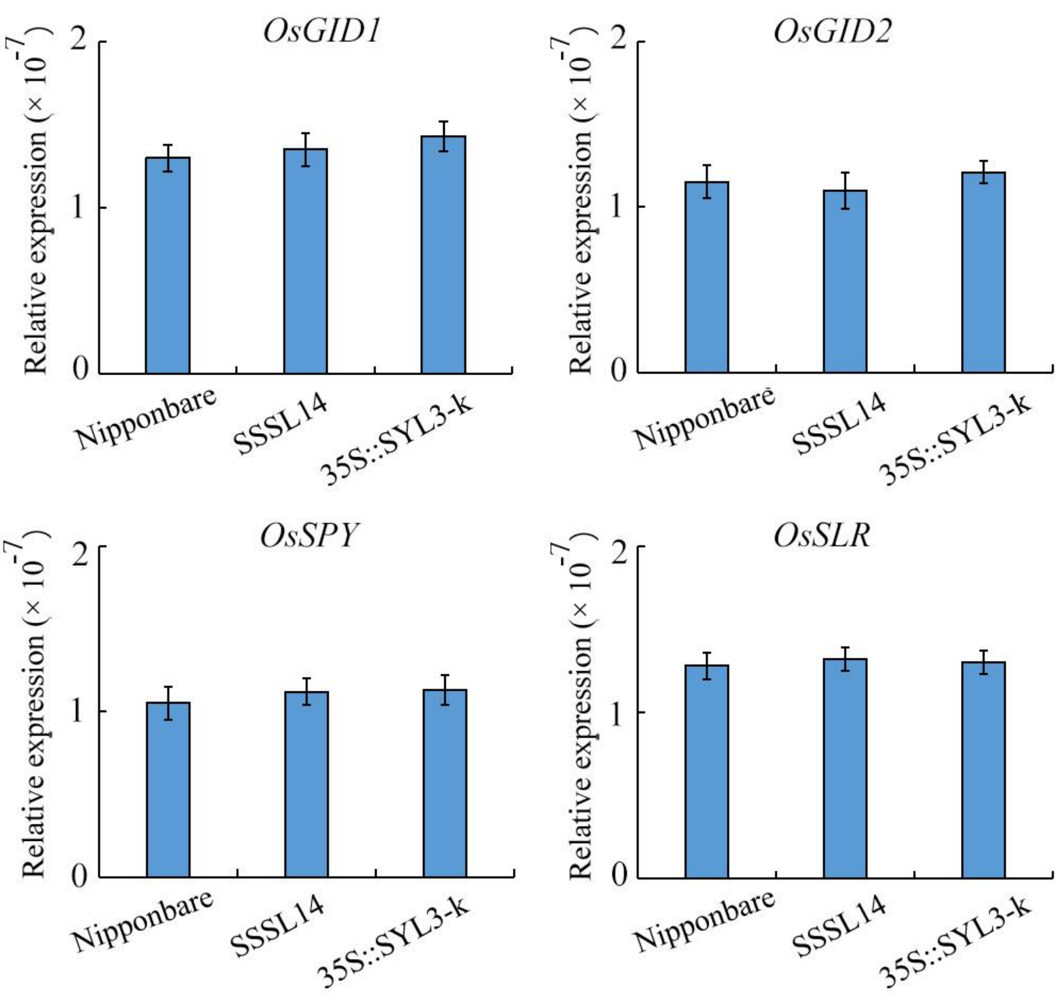

Figure S5 qRT-PCR analysis of genes associated with GA signal transduction pathway in Nipponbare, SSSL14 and transgenic 35S::SYL3-k overexpression line. The relative expression level of each gene in pistil at stage 8 of young panicle differentiation of Nipponbare, SSSL14 and transgenic 35S::SYL3-k overexpression line were analyzed by qRT-PCR and normalized using the ubiquitin gene as an internal control. Data are means ± SD (n=3). Asterisks indicate statistically significant differences compared with the wild type at P <0.01 by Student's *t* test.

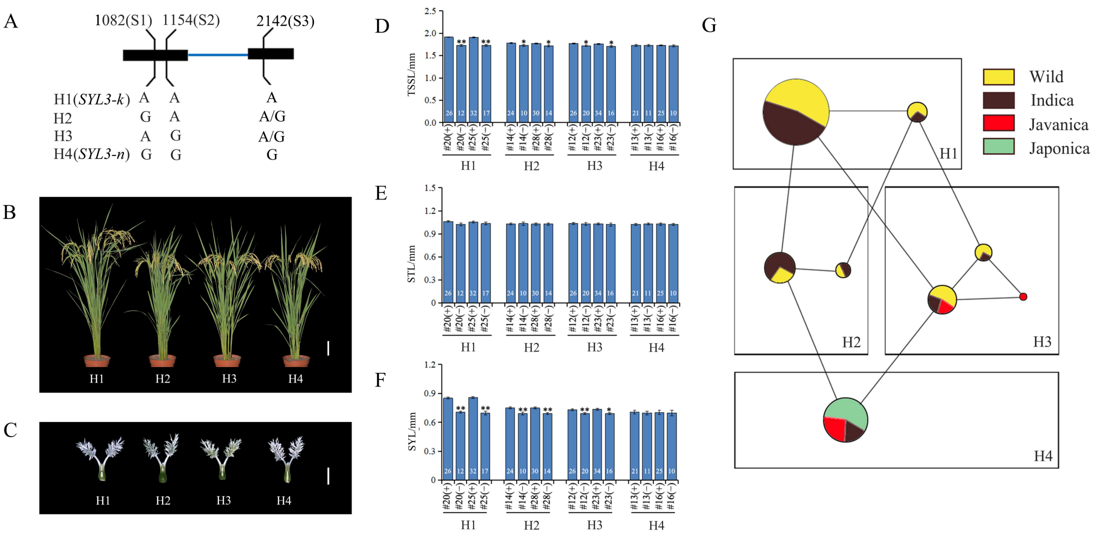

Figure S6 Natural variation and network analysis for *SYL3*. (A) A diagram showed the four transgenic constructs representing four haplotypes based on the two polymorphic sites (S1 and S2). The black boxes denote exons and the blue line denotes introns. (B-F) Plant morphology (B), pistil morphology (C), TSSL (D), STL (E) and SYL (F) of the transgenic plants harboring the four haplotypes. Two independent T_1 transgenic families for each transgene (#20 and #25 for H1, #14 and #28 for H2, #12 and #43 for H3, #14 and #17 for H4) were showed. Plus (+) and minus (-) symbols indicate transgene-positive and transgene-negative segregates, respectively. Numbers of the samples used in the study are indicated in the columns. Data represent means ± SD. * P<0.05, ** *P*<0.01, Student's *t*-test. (G) Allele network of *SYL3* in 136 rice accessions. Four groups (H1, H2, H3 and H4) of alleles were identified, with each group consisting of multiple allele subtypes based on the SNPs uncovered in the introns of *SYL3*. The allele frequencies are proportional to the size of the circles. The length of the lines between the circles indicates the evolutionary distance. The proportions for wild, *indica*, *javanica* and *japonica* rice are represented by different colors, respectively. TSSL, the sum of stigma and style length; STL, stigma length; SYL, style length.

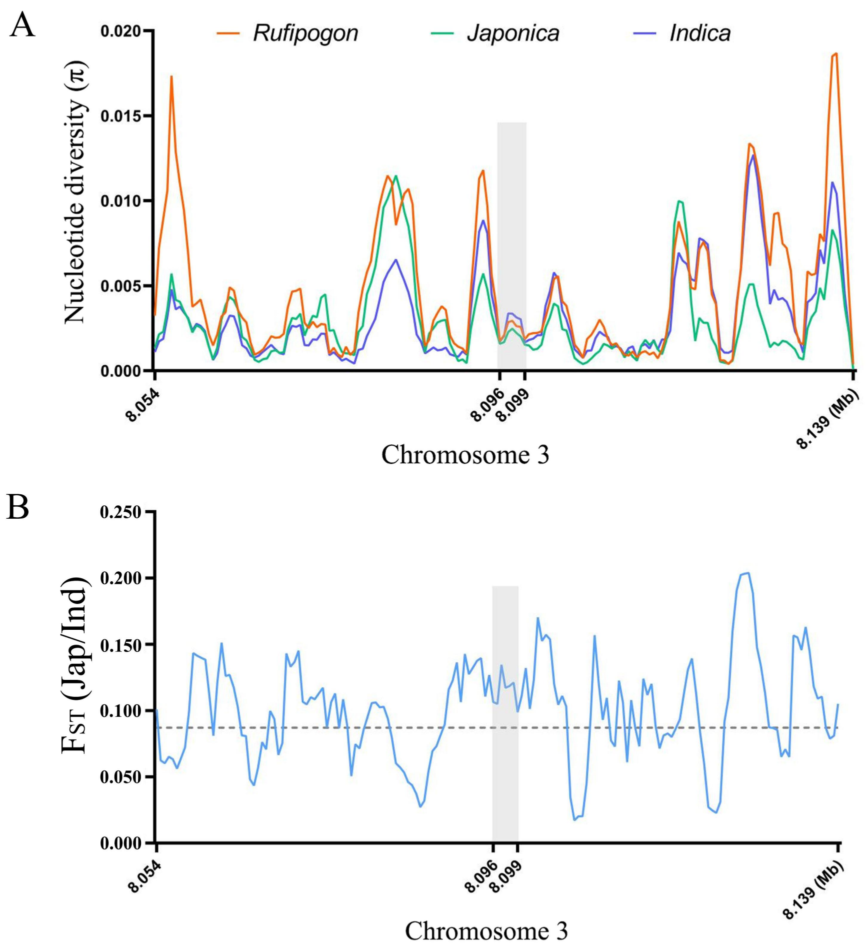

Figure S7 π values (A) and F_{ST} values (B) between indica and japonica in the 85 kb genomic region containing the *SYL3* locus (the shadow column). The dashed line indicates the genome-wide threshold.
