# Supplementary material for: SYL3-k increases style length and yield of F1 seeds via enhancement of endogenous GA4 content in Oryza sativa L. pistils
Source: Theor Appl Genet. 2021 Oct 17;135(1):321–36. doi: 10.1007/s00122-021-03968-y (PMC8741667; doi:10.1007/s00122-021-03968-y)
Supplement: Supplementary file 2 — Supplementary file2 (DOCX 26 KB) [file 122_2021_3968_MOESM2_ESM.docx]

**Table S1** The accessions list and SNPs of *SYL3* in cultivated and wild rice.

| Taxon | Accession name | Accession ID | Country | latitude | longitude | S1 | S2 | S3 | Allele | STL/mm | SYL/mm | TSSL/mm |
| --- | --- | --- | --- | --- | --- | --- | --- | --- | --- | --- | --- | --- |
| *O*. rufipogon | Puye-1 | **S6094** | China | N23° | E113° | A | A | G | H1 | 1.368 | 0.62 | 1.988 |
| (Perennial, 19) | Puye-2 | **S6146** | China | N23° | E113° | G | A | G | H2 | ns | ns | ns |
|  | Puye-3 | **S6162** | China | N23° | E113° | G | A | G | H2 | 1.201 | 0.804 | 2.005 |
|  | Puye-4 | **S6191** | China | N23° | E113° | G | A | G | H2 | 1.358 | 0.872 | 2.23 |
|  | Puye-5 | **S7992** | China | N23° | E115° | G | A | G | H2 | ns | ns | ns |
|  | Puye-6 | **S01196** | China | N28° | E116° | G | A | G | H2 | 1.399 | 0.745 | 2.144 |
|  | Yaoye-1 | **0553** | China | N23° | E112° | A | G | G | H3 | 1.209 | 0.758 | 1.967 |
|  | Yaoye-2 | **0551** | China | N23° | E112° | A | G | G | H3 | 1.34 | 0.868 | 2.208 |
|  | Puye-7 | **S1067** | China | N19° | E109° | A | G | G | H3 | 1.251 | 0.679 | 1.93 |
|  | Yaoye-3 | **2015102203** | China | N23° | E111° | A | G | G | H3 | 1.159 | 0.763 | 1.922 |
|  | Yaoye-4 | **2015092305** | China | N23° | E112° | A | G | G | H3 | 1.232 | 0.677 | 1.909 |
|  | Puye-8 | **S9032** | China | N23° | E113° | G | A | G | H2 | ns | ns | ns |
|  | Puye-9 | **S3007** | China | N22° | E112° | G | A | G | H2 | 1.142 | 0.834 | 1.976 |
|  | Puye-10 | **S2282** | China | N21° | E110° | G | A | G | H2 | 1.308 | 0.73 | 2.038 |
|  | Puye-11 | **S8091** | China | N22° | E115° | A | G | G | H3 | ns | ns | ns |
|  | Puye-12 | **S7993** | China | N23° | E113° | A | G | G | H3 | 1.251 | 0.879 | 2.13 |
|  | Puye-13 | **S2525** | China | N21° | E111° | A | G | G | H3 | 1.182 | 0.76 | 1.942 |
|  | Puye-14 | **S8023** | China | N22° | E115° | G | A | G | H2 | 1.252 | 0.749 | 2.001 |
|  | Puye-15 | **S8016** | China | N22° | E115° | G | A | G | H2 | 1.243 | 0.7 | 1.943 |
| *O*. rufipogon | Gaoganye | **E1-1** | Brazil | S15° | W47° | G | A | G | H2 | ns | ns | ns |
| (annual, 16) | Niwalaye-1 | **E14-1** | Myanmar | N19° | E96° | G | A | G | H2 | ns | ns | ns |
|  | Youye-1 | **M3** | China | N19° | E108° | A | A | G | H1 | ns | ns | ns |
|  | Youye-2 | **M4** | China | N19° | E109° | A | A | G | H1 | ns | ns | ns |
|  | Youye-3 | **M64** | China | N18° | E109° | G | A | G | H2 | ns | ns | ns |
|  | Youye-4 | **M37** | China | N18° | E109° | A | G | G | H3 | ns | ns | ns |
|  | Zhanyingye | **E8-2** | Cuba | N23° | W82° | A | A | G | H1 | ns | ns | ns |
|  | Kuoye-1 | **E-10** | Mexico | N19° | W99° | A | A | G | H1 | ns | ns | ns |
|  | Kuoye-2 | **E9-27⑥** | Bolivia | S16° | W68° | A | A | G | H1 | ns | ns | ns |
|  | Niwalaye-2 | **E14-012** | Sri Lanka | N6° | E79° | A | G | G | H3 | ns | ns | ns |
|  | Bandianye | **E16-21** | Ghana | N5° | E0° | A | G | G | H3 | ns | ns | ns |
|  | Maye-1 | **E19-2** | Philippines | N14° | E120° | A | G | G | H3 | ns | ns | ns |
|  | Maye-1 | **E19-5** | India | N28° | E77° | G | A | G | H2 | ns | ns | ns |
|  | Puye-16 | **E18-22** | Sri Lanka | N6° | E79° | A | G | G | H3 | ns | ns | ns |
|  | Puye-17 | **E18-12** | Myanmar | N19° | E96° | G | A | G | H2 | ns | ns | ns |
|  | Youye-5 | **M2** | China | N19° | E109° | A | G | G | H3 | ns | ns | ns |
| *O. sativa* ssp. | Qimiaoxiang 3hao | H1235 | China | N28° | E112° | G | G | G | H4 | 1.31 | 0.6 | 1.92 |
| *Indica* (44) | IR112 | H1316 | Philippines | N14° | E120° | G | G | G | H4 | 1.21 | 0.89 | 2.1 |
|  | IR36 | H1318 | Philippines | N14° | E120° | G | G | G | H4 | 1.18 | 0.82 | 2 |
|  | Yuedao 119 | H1194 | Vietnam | N21° | E105° | G | G | G | H4 | 1.36 | 0.69 | 2.05 |
|  | Dholi Boro | H1162 | Bangladesh | N23° | E90° | A | A | G | H1 | 1.33 | 0.57 | 1.91 |
|  | BJ1 | *23820* | Sri Lanka | N6° | E79° | A | A | A | H1 | 1.54 | 0.59 | 2.12 |
|  | DV85 | H1322 | Bangladesh | N23° | E90° | A | A | G | H1 | 1.64 | 0.63 | 2.27 |
|  | Huanghuazhan | ES2007017 | China | N28° | E112° | G | G | G | H4 | 1.34 | 0.82 | 2.15 |
|  | IR68275B | H1223 | Philippines | N14° | E120° | G | G | G | H4 | 1.13 | 0.8 | 1.93 |
|  | IR20 | H1225 | Philippines | N14° | E120° | G | G | G | H4 | 1.27 | 0.87 | 2.14 |
|  | IR26 | H1226 | Philippines | N14° | E120° | G | G | G | H4 | 1.13 | 0.73 | 1.86 |
|  | Xiangwanxian 3hao | XS1996096 | China | N29° | E113° | G | G | G | H4 | 1.3 | 0.7 | 2.01 |
|  | Zhenpin B | H1242 | China | N28° | E115° | G | G | G | H4 | 1.39 | 0.96 | 2.35 |
|  | 256B | H1243 | China | N28° | E115° | G | G | G | H4 | 1.24 | 0.63 | 1.87 |
|  | Taizhongxianxuan 220 | *30-00244* | China | N24° | E120° | G | G | G | H4 | 1.47 | 0.75 | 2.22 |
|  | IR112-12 | H1245 | Philippines | N14° | E120° | G | G | G | H4 | 1.22 | 0.75 | 1.98 |
|  | Shufeng 101 | H1246 | China | N30° | E104° | G | G | G | H4 | 1.63 | 0.76 | 2.38 |
|  | JC92 | H1248 | Philippines | N14° | E120° | G | G | G | H4 | 1.16 | 0.88 | 2.04 |
|  | II-32B | H1274 | China | N28° | E112° | G | G | G | H4 | 1.45 | 0.6 | 2.05 |
|  | Fanhaopi | *21-03879* | China | N24° | E102° | A | A | A | H1 | 1.49 | 0.65 | 2.14 |
|  | Erjiunan 1hao | *ZD-00474* | China | N30° | E120° | A | G | G | H3 | 1.36 | 0.53 | 1.89 |
|  | Nongxiang 32 | XS2015009 | China | N28° | E112° | A | A | G | H1 | 1.48 | 0.87 | 2.36 |
|  | Xiangyaxiangzhen | SS2013030 | China | N28° | E112° | A | A | G | H1 | 1.24 | 0.63 | 1.87 |
|  | Malaihong-2 | H1312 | China | N23° | E113° | A | G | G | H3 | 1.34 | 0.62 | 1.95 |
|  | Ziyeying | H1313 | China | N23° | E113° | A | G | G | H3 | 1.27 | 0.61 | 1.88 |
|  | IR-44595 | H1317 | Philippines | N14° | E120° | A | A | G | H1 | 1.49 | 0.77 | 2.26 |
|  | Taifeng B | H1321 | China | N23° | E113° | A | A | G | H1 | 1.73 | 0.72 | 2.44 |
|  | Huangyuezhan | YS2008037 | China | N23° | E113° | A | A | G | H1 | 1.27 | 0.65 | 1.92 |
|  | Zhennuo 1hao | YS2006015 | China | N23° | E113° | A | A | G | H1 | 1.19 | 0.77 | 1.96 |
|  | Chuan 29B | H1324 | China | N30° | E104° | G | G | G | H4 | 1.39 | 0.69 | 2.08 |
|  | Lvdao Q7 | ES2014004 | China | N30° | E114° | A | A | G | H1 | 1.11 | 0.84 | 1.95 |
|  | R608 | H1327 | China | N28° | E112° | A | A | G | H1 | 1.27 | 0.8 | 2.07 |
|  | R862 | H1328 | China | N28° | E112° | A | A | G | H1 | 1.65 | 0.84 | 2.49 |
|  | Wushanximiao | YS2009031 | China | N23° | E113° | A | A | G | H1 | 1.25 | 0.68 | 1.93 |
|  | Yuejingsimiao 2hao | YS2006067 | China | N23° | E113° | A | A | G | H1 | 1.24 | 0.61 | 1.84 |
|  | R7954 | H1334 | China | N30° | E120° | A | A | G | H1 | 1.34 | 0.72 | 2.06 |
|  | Qing 2 | H1336 | China | N33° | E120° | G | A | G | H2 | 1.16 | 0.75 | 1.9 |
|  | Qing 3 | H1337 | China | N33° | E120° | G | A | G | H2 | 1.36 | 0.7 | 2.06 |
|  | Qing 4 | H1338 | China | N33° | E120° | A | A | G | H1 | 1.4 | 0.89 | 2.29 |
|  | Qing 6 | H1340 | China | N33° | E120° | A | A | G | H1 | 1.27 | 0.88 | 2.15 |
|  | Qing 7 | H1341 | China | N33° | E120° | A | A | G | H1 | 1.15 | 0.74 | 1.9 |
|  | Qing 8 | H1342 | China | N33° | E120° | A | A | G | H1 | 1.3 | 0.75 | 2.05 |
|  | Qing 9 | H1343 | China | N33° | E120° | G | A | G | H2 | 1.27 | 0.71 | 1.99 |
|  | Qing 10 | H1344 | China | N33° | E120° | A | A | G | H1 | 1.42 | 0.8 | 2.22 |
| *O.sativa* ssp. | Nanton 53 | H1600 | Thaailand | N13° | E100° | G | G | G | H4 | 1.31 | 0.7 | 2.01 |
| *Javanica* (20) | Ketan Nangka | *16466* | Indonesia | S6° | E106° | G | G | G | H4 | 1.15 | 0.49 | 1.64 |
|  | Vary Lava | *11033* | Malaysia | N3° | E101° | G | G | G | H4 | 1.34 | 0.66 | 2 |
|  | Niaw Ma-dan | *16787* | Vietnam | N21° | E105° | G | G | G | H4 | 1.22 | 0.61 | 1.83 |
|  | DJAWA BLAWU | *16379* | Indonesia | S6° | E106° | A | G | G | H3 | 1.17 | 0.44 | 1.61 |
|  | TAI-4 | *69056* | Malaysia | N3° | E101° | A | G | G | H3 | 1.23 | 0.63 | 1.86 |
|  | Qutube-n | H1520 | Malaysia | N3° | E101° | G | G | G | H4 | 1.16 | 0.68 | 1.84 |
|  | Arias | H1178 | Indonesia | S6° | E106° | G | G | G | H4 | 1.09 | 0.73 | 1.82 |
|  | CAMOR | *10861* | Indonesia | S6° | E106° | G | G | G | H4 | 1.12 | 0.77 | 1.89 |
|  | Gendjah Gempol | *12483* | Indonesia | S6° | E106° | G | G | G | H4 | 1.4 | 0.72 | 2.12 |
|  | BuluH BAWU | *16481* | Indonesia | S6° | E106° | G | G | G | H4 | 1.07 | 0.99 | 2.06 |
|  | SIPAK | *23182* | Indonesia | S6° | E106° | G | G | G | H4 | 0.96 | 0.69 | 1.65 |
|  | GUNDIL PUTIH | *67205* | Indonesia | S6° | E106° | G | G | G | H4 | 1.24 | 0.86 | 2.1 |
|  | PADI BULU | *67235* | Indonesia | S6° | E106° | G | G | G | H4 | 1.11 | 0.61 | 1.71 |
|  | GUNDIL KUNING | *67250* | Indonesia | S6° | E106° | G | G | G | H4 | 1.1 | 0.58 | 1.68 |
|  | BULU POTE | *73989* | Indonesia | S6° | E106° | G | G | G | H4 | 0.9 | 0.64 | 1.55 |
|  | Mack Hing | *77230* | Indonesia | S6° | E106° | G | G | G | H4 | 1.15 | 0.62 | 1.76 |
|  | RODJOLELE | *9909* | Indonesia | S6° | E106° | G | G | G | H4 | 1.49 | 0.56 | 2.04 |
|  | BOEGI IMBA | *4166* | Indonesia | S6° | E106° | G | G | G | H4 | 0.98 | 0.65 | 1.64 |
|  | BOEGI BOERA | *41665* | Indonesia | S6° | E106° | G | G | G | H4 | 1.39 | 0.74 | 2.13 |
| *O.sativa* ssp. | Yueguang | H1200 | Japan | N35° | E139° | G | G | G | H4 | 0.99 | 0.57 | 1.55 |
| *Japonica* (37) | Yimuhu | H1202 | Japan | N35° | E139° | G | G | G | H4 | 1.05 | 0.5 | 1.55 |
|  | RM123 | H1206 | Japan | N35° | E139° | G | G | G | H4 | 0.95 | 0.46 | 1.41 |
|  | RT60 | H1207 | Japan | N35° | E139° | G | G | G | H4 | 1.04 | 0.63 | 1.67 |
|  | SH189 | H1210 | Japan | N35° | E139° | G | G | G | H4 | 1.09 | 0.52 | 1.6 |
|  | Xudao 25-7 | H1215 | China | N34° | E117° | G | G | G | H4 | 1.09 | 0.52 | 1.61 |
|  | Xudao 25-8 | H1216 | China | N34° | E117° | G | G | G | H4 | 1 | 0.48 | 1.47 |
|  | Gaoxiong 139 | H1240 | China | N22° | E120° | G | G | G | H4 | 1.07 | 0.56 | 1.63 |
|  | Hongyin 1 | H1253 | China | N32° | E118° | G | G | G | H4 | 1.03 | 0.54 | 1.57 |
|  | Hongyin 2 | H1254 | China | N32° | E118° | G | G | G | H4 | 1.03 | 0.58 | 1.6 |
|  | Yaxuenuo | H1160 | China | N32° | E118° | G | G | G | H4 | 0.98 | 0.58 | 1.56 |
|  | Yandao 6hao | SS200205 | China | N33° | E120° | G | G | G | H4 | 1.16 | 0.51 | 1.67 |
|  | Wandao 68 | WPS03010384 | China | N31° | E117° | G | G | G | H4 | 0.96 | 0.45 | 1.42 |
|  | Xiangjing 9407 | LS200216 | China | N32° | E118° | G | G | G | H4 | 1.03 | 0.56 | 1.59 |
|  | Zhongjing 212 | WS891061 | China | N40° | E111° | G | G | G | H4 | 1.02 | 0.46 | 1.48 |
|  | Zhongjing 9677 | H1124 | China | N40° | E111° | G | G | G | H4 | 0.9 | 0.53 | 1.42 |
|  | Zhenghan 2hao | GS2003031 | China | N34° | E113° | G | G | G | H4 | 0.86 | 0.6 | 1.46 |
|  | RD21 | H1530 | China | N34° | E113° | G | G | G | H4 | 1.19 | 0.52 | 1.71 |
|  | Koshihikari | H1164 | Japan | N35° | E139° | G | G | G | H4 | 1.18 | 0.46 | 1.63 |
|  | Yangfujing 8hao | SS200608 | China | N32° | E119° | G | G | G | H4 | 1.05 | 0.58 | 1.63 |
|  | Lianjing 9823 | *ZD-05639* | China | N34° | E119° | G | G | G | H4 | 0.99 | 0.52 | 1.51 |
|  | Huifeng 1 | H1508 | China | N33° | E120° | G | G | G | H4 | 1.05 | 0.59 | 1.64 |
|  | Yandao 8hao | SS200307 | China | N33° | E120° | G | G | G | H4 | 0.66 | 0.69 | 1.35 |
|  | Mudanjiang 29 | HS2006007 | China | N44° | E129° | G | G | G | H4 | 1.01 | 0.54 | 1.55 |
|  | Songjing 10 | HS2005005 | China | N44° | E129° | G | G | G | H4 | 0.91 | 0.6 | 1.51 |
|  | Dongnong 424 | HS2005002 | China | N45° | E126° | G | G | G | H4 | 1 | 0.55 | 1.55 |
|  | Longnuo 3hao | HS2009015 | China | N45° | E126° | G | G | G | H4 | 0.86 | 0.55 | 1.41 |
|  | Longjing 27 | HS2009010 | China | N45° | E126° | G | G | G | H4 | 0.94 | 0.47 | 1.41 |
|  | Longjing 25 | HS2009009 | China | N45° | E126° | G | G | G | H4 | 1.06 | 0.44 | 1.5 |
|  | Longjing 22 | HS2008010 | China | N45° | E126° | G | G | G | H4 | 0.92 | 0.47 | 1.39 |
|  | Longjing 20 | HS2007004 | China | N45° | E126° | G | G | G | H4 | 0.9 | 0.5 | 1.4 |
|  | Longjing 17 | HS2007001 | China | N45° | E126° | G | G | G | H4 | 0.99 | 0.66 | 1.65 |
|  | Longjing 15 | HS2006001 | China | N45° | E126° | G | G | G | H4 | 1.05 | 0.59 | 1.64 |
|  | Longdao 8hao | HS2008019 | China | N45° | E126° | G | G | G | H4 | 0.97 | 0.58 | 1.55 |
|  | Sihao 4330 | H1080 | China | N33° | E118° | G | G | G | H4 | 1.02 | 0.52 | 1.55 |
|  | Ludao | H1081 | China | N32° | E118° | G | G | G | H4 | 1.08 | 0.67 | 1.75 |
|  | Shengtangqing | H1082 | China | N31° | E120° | G | G | G | H4 | 1.06 | 0.61 | 1.67 |

Note: In column “Accession ID”, the word with Bold means that the accessions were provided by Guangdong Academy of Agricultural Sciences; the word with Italics means that these accessions were provided by Rice germplasm resource center in Beijing; the word with underline means that these accessions were obtained from National rice data center in Hangzhou; the remaining accessions were saved by the corresponding authors. “ns” represents a lack of a value. STL, stigma length; SYL, style length; TSSL, the sum of stigma and style length.
