# Supplementary material for: SYL3-k increases style length and yield of F1 seeds via enhancement of endogenous GA4 content in Oryza sativa L. pistils
Source: Theor Appl Genet. 2021 Oct 17;135(1):321–36. doi: 10.1007/s00122-021-03968-y (PMC8741667; doi:10.1007/s00122-021-03968-y)
Supplement: Supplementary file 3 — Supplementary file3 (DOCX 14 KB) [file 122_2021_3968_MOESM3_ESM.docx]

**Table S2** Primers used for vector construction, quantitative RT-PCR and sequencing.

| Primer name | Forward primer (5’-3’) | Reverse Primer (5’-3’) | Description | Reference |
| --- | --- | --- | --- | --- |
| *SYL3*-full genome | cagtGGTCTCaagctcttgaatttgttaagtatct | cagtGGTCTCagatcgatttgatggtattaccaaa | Complementation test | This study |
| Overexpression | cagtGGTCTCacaaccttgaatttgttaagtatct | cagtGGTCTCatacatcagccccaatccaaaccaa | Overexpression test | This study |
| *SYL3-*RFP | cagtGGTCTCacaacatggtgaagtgtagggcacg | cagtGGTCTCatacagccccaatccaaaccaaaga | Subcellular location | This study |
| *SYL3*-GUS | cagtGGTCTCatagatgtcattgtcatagggtgac | cagtGGTCTCagttggatttgatggtattaccaaa | GUS vector construct | This study |
| *qRT-Ubiquitin* | ACCCTGGCTGACTACAACATC | AGTTGACAGCCCTAGGGTG | Quantitative RT-PCR | Known sequence |
| *qRT-SYL3* | GCGCCACACTACCATCTTCA | CCCGCTTTGGGTTGAGCTA | Quantitative RT-PCR | Liu et al., 2015 |
| H2-1 | gagtGGTCTCacaaccttgaatttgttaagtatctcat | gagtGGTCTCagttctagttcacggatttccaca | H2 construct | This study |
| H2-2 | gagtCACCTGCaaaagaacgtgaagaaagagagttgaaagag | gagtCACCTGCaaaatacatcagccccaatccaaaccaaaga | H2 construct | This study |
| H3-1 | gagtGGTCTCacaaccttgaatttgttaagtatctcat | gagtGGTCTCactattgcctcccgcagaagctta | H3 construct | This study |
| H3-2 | gagtCACCTGCaaaatagcagcaagggatggagggcaattg | gagtCACCTGCaaaatacatcagccccaatccaaaccaaaga | H3 construct | This study |
| *SYL3*-seq-1 | TCCTCCTCTCACCAGACCTT | GCTGATACATAGGCAAGCTGT | Sequencing *SYL3* coding region in rice accessions | This study |
| *SYL3*-seq-2 | GTCTCCGTGTGTTGTTTAGTACA | CGCAAAGCGGTGAAAACTGA | Sequencing *SYL3* coding region in rice accessions | This study |
