# Supplementary material for: SYL3-k increases style length and yield of F1 seeds via enhancement of endogenous GA4 content in Oryza sativa L. pistils
Source: Theor Appl Genet. 2021 Oct 17;135(1):321–36. doi: 10.1007/s00122-021-03968-y (PMC8741667; doi:10.1007/s00122-021-03968-y)
Supplement: Supplementary file 4 — Supplementary file4 (DOCX 14 KB) [file 122_2021_3968_MOESM4_ESM.docx]

**Table S3** Primers used in sequencing the 20 genes surrounding *SYL3*.

| Code | Locus ID | Position / bp | Forward primer sequence (5’-3’) | Reverse primer sequence (5’-3’) |
| --- | --- | --- | --- | --- |
| 1 | Os03t0251425 (RAP-DB) | 7,980,614-7,982,643 | AGCATGTTTCTGGGCCATGT | CCCTCTCTCGGCGAATTTGT |
| 2 | Os03t0251700 (RAP-DB) | 7.988,159-8,000,400 | GGGCAGGTAGTACACACGAC | CTGAAACTGAGACCTTTCCCCC |
| 3 | Os03t0251950 (RAP-DB) | 8,004,062-8,004,729 | AGTCGAGGCGAAAATCTGGG | GTGGCTTACAGTTGCTGATGC |
| 4 | Os03t0252100 (RAP-DB) | 8,010,885-8,012,566 | CGGGGAGACGGCAGAATTTA | GTCGGCTCCCTCATACAGTC |
| 5 | LOC_Os03g14780 | 8,033,545-8,033,930 | GGGGATCGGAATTAACCCGC | GAGCTGGGAGGGAACAGTTG |
| 6 | LOC_Os03g14800 | 8,046,989-8,052,547 | GACGTGTTATGGCGCTTGTT | CAACGTGCAAGCATAACGGA |
| 7 | LOC_Os03g14810 | 8,061,295-8,061888 | ATCCAAAGCCGTAGACCACA | TCATCTTTAGCAAGTCGCCTCT |
| 8 | LOC_Os03g14820 | 8,064,513-8,065,253 | ATTTCACCCGTAGGGTCGG | GACAAGCTAGCCATTGCCG |
| 9 | Os03G0253100 (RAP-DB) | 8,067,630-8,071,826 | GGGACATTCCTTGACCCTGG | TTTTGCCGCCTCTCCTTTCT |
| 10 | LOC_Os03g14840 | 8,087,161-8,088,952 | ATCTGACCGTTGGATTGCCC | AGCTCAACCACTCATTCAGCA |
| 11 | LOC_Os03g14860 | 8,101,572-8,102,858 | GGTAGACAATGCTGGCTCCT | GGGAGACTTCTCTTCGCCG |
| 12 | LOC_Os03g14880 | 8,106,337-8,108,381 | TTCAGACAATGCACGAGGCT | CAGAGCAAGGGCAATCATGG |
| 13 | LOC_Os03g14890 | 8,111,990-8,117,742 | AATCGCTGGTCAGGGGAATG | AATAGCGGTACTGCGAAGGG |
| 14 | LOC_Os03g14900 | 8,122,245-8,126,084 | ATAGCCAACCCAGCCTGCTA | CGATTCGCAGGCTTTCCTCT |
| 15 | LOC_Os03g14910 | 8,130,192-8,131,506 | ATTGTCAGCCACAAACCACTG | TTCTTGTTCCATTTCTGTTCGGG |
| 16 | LOC_Os03g14915 | 8,132,532-8,134,227 | AAACACTGGTTCGCCCAACT | AACGGGAGCAAAGACGTTGT |
| 17 | LOC_Os03g14920 | 8,135,014-8,136,274 | ATGGCCCAAATCGGATGGTC | AGGGCGCAACATTACTGCAT |
| 18 | LOC_Os03g14950 | 8,152,544-8,154,731 | TGTTTAATCACCCCGCCGAA | ATCGCCGTTGCATCCACTTA |
| 19 | Os03t0255000 (RAP-DB) | 8,191,896-8,195,080 | CCCAGCCATGTCTCTAGTCG | CACACTTTTGATGTACCGGGTG |
| 20 | Os03g0255200 (RAP-DB) | 8,207,583-8,211,913 | AAGCCGCACTCCAGGAAAAT | ATGCATAGGTGTGGTCAATTCC |
