# Supplementary material for: SYL3-k increases style length and yield of F1 seeds via enhancement of endogenous GA4 content in Oryza sativa L. pistils
Source: Theor Appl Genet. 2021 Oct 17;135(1):321–36. doi: 10.1007/s00122-021-03968-y (PMC8741667; doi:10.1007/s00122-021-03968-y)
Supplement: Supplementary file 5 — Supplementary file5 (DOCX 15 KB) [file 122_2021_3968_MOESM5_ESM.docx]

**Table S4** Summary for parameters (*K, d, Nb*) used in the coalescent simulation.

| *k* | *d* | *Nb* |  | *k* | *d* | *Nb* |  | *k* | *d* | *Nb* |  | *k* | *d* | *Nb* |
| --- | --- | --- | --- | --- | --- | --- | --- | --- | --- | --- | --- | --- | --- | --- |
| 0.0001 | 200 | 0.02 |  | 0.10 | 200 | 20 |  | 0.35 | 200 | 70 |  | 0.60 | 200 | 120 |
| 0.0001 | 500 | 0.05 |  | 0.10 | 500 | 50 |  | 0.35 | 500 | 175 |  | 0.60 | 500 | 300 |
| 0.0001 | 1000 | 0.1 |  | 0.10 | 1000 | 100 |  | 0.35 | 1000 | 350 |  | 0.60 | 1000 | 600 |
| 0.0001 | 1500 | 0.15 |  | 0.10 | 1500 | 150 |  | 0.35 | 1500 | 525 |  | 0.60 | 1500 | 900 |
| 0.0001 | 2000 | 0.2 |  | 0.10 | 2000 | 200 |  | 0.35 | 2000 | 700 |  | 0.60 | 2000 | 1200 |
| 0.0001 | 3000 | 0.3 |  | 0.10 | 3000 | 300 |  | 0.35 | 3000 | 1050 |  | 0.60 | 3000 | 1800 |
| 0.001 | 200 | 0.2 |  | 0.15 | 200 | 30 |  | 0.40 | 200 | 80 |  | 0.65 | 200 | 130 |
| 0.001 | 500 | 0.5 |  | 0.15 | 500 | 75 |  | 0.40 | 500 | 200 |  | 0.65 | 500 | 325 |
| 0.001 | 1000 | 1 |  | 0.15 | 1000 | 150 |  | 0.40 | 1000 | 400 |  | 0.65 | 1000 | 650 |
| 0.001 | 1500 | 1.5 |  | 0.15 | 1500 | 225 |  | 0.40 | 1500 | 600 |  | 0.65 | 1500 | 975 |
| 0.001 | 2000 | 2 |  | 0.15 | 2000 | 300 |  | 0.40 | 2000 | 800 |  | 0.65 | 2000 | 1300 |
| 0.001 | 3000 | 3 |  | 0.15 | 3000 | 450 |  | 0.40 | 3000 | 1200 |  | 0.65 | 3000 | 1950 |
| 0.005 | 200 | 1 |  | 0.20 | 200 | 40 |  | 0.45 | 200 | 90 |  | 0.70 | 200 | 140 |
| 0.005 | 500 | 2.5 |  | 0.20 | 500 | 100 |  | 0.45 | 500 | 225 |  | 0.70 | 500 | 350 |
| 0.005 | 1000 | 5 |  | 0.20 | 1000 | 200 |  | 0.45 | 1000 | 450 |  | 0.70 | 1000 | 700 |
| 0.005 | 1500 | 7.5 |  | 0.20 | 1500 | 300 |  | 0.45 | 1500 | 675 |  | 0.70 | 1500 | 1050 |
| 0.005 | 2000 | 10 |  | 0.20 | 2000 | 400 |  | 0.45 | 2000 | 900 |  | 0.70 | 2000 | 1400 |
| 0.005 | 3000 | 15 |  | 0.20 | 3000 | 600 |  | 0.45 | 3000 | 1350 |  | 0.70 | 3000 | 2100 |
| 0.01 | 200 | 2 |  | 0.25 | 200 | 50 |  | 0.50 | 200 | 100 |  | 0.75 | 200 | 150 |
| 0.01 | 500 | 5 |  | 0.25 | 500 | 125 |  | 0.50 | 500 | 250 |  | 0.75 | 500 | 375 |
| 0.01 | 1000 | 10 |  | 0.25 | 1000 | 250 |  | 0.50 | 1000 | 500 |  | 0.75 | 1000 | 750 |
| 0.01 | 1500 | 15 |  | 0.25 | 1500 | 375 |  | 0.50 | 1500 | 750 |  | 0.75 | 1500 | 1125 |
| 0.01 | 2000 | 20 |  | 0.25 | 2000 | 500 |  | 0.50 | 2000 | 1000 |  | 0.75 | 2000 | 1500 |
| 0.01 | 3000 | 30 |  | 0.25 | 3000 | 750 |  | 0.50 | 3000 | 1500 |  | 0.75 | 3000 | 2250 |
| 0.05 | 200 | 10 |  | 0.30 | 200 | 60 |  | 0.55 | 200 | 110 |  | 0.80 | 200 | 160 |
| 0.05 | 500 | 25 |  | 0.30 | 500 | 150 |  | 0.55 | 500 | 275 |  | 0.80 | 500 | 400 |
| 0.05 | 1000 | 50 |  | 0.30 | 1000 | 300 |  | 0.55 | 1000 | 550 |  | 0.80 | 1000 | 800 |
| 0.05 | 1500 | 75 |  | 0.30 | 1500 | 450 |  | 0.55 | 1500 | 825 |  | 0.80 | 1500 | 1200 |
| 0.05 | 2000 | 100 |  | 0.30 | 2000 | 600 |  | 0.55 | 2000 | 1100 |  | 0.80 | 2000 | 1600 |
| 0.05 | 3000 | 150 |  | 0.30 | 3000 | 900 |  | 0.55 | 3000 | 1650 |  | 0.80 | 3000 | 2400 |

The parameter *d*, the duration of the bottleneck; the parameter *Nb* were used for bottleneck. The *K*, that is the ration of *Nb* and *d*, was used to describe the severity of the bottleneck in domestication.
