# Supplementary material for: SYL3-k increases style length and yield of F1 seeds via enhancement of endogenous GA4 content in Oryza sativa L. pistils
Source: Theor Appl Genet. 2021 Oct 17;135(1):321–36. doi: 10.1007/s00122-021-03968-y (PMC8741667; doi:10.1007/s00122-021-03968-y)
Supplement: Supplementary file 6 — Supplementary file6 (DOCX 14 KB) [file 122_2021_3968_MOESM6_ESM.docx]

**Table S5** The Pearson correlation coefficients between three SNPs and pistil traits.

| Traits | SNP Position | | | | | | | |
| --- | --- | --- | --- | --- | --- | --- | --- | --- |
|  | S1 | |  | S2 | |  | S3 | |
|  | *P* value | *r* |  | *P* value | *r* |  | *P* value | *r* |
| Stigma length | 0.163 | 0.120 |  | 0.068 | 0.157 |  | 0.098 | 0.142 |
| Style length | 0.039 | 0.177* |  | 0.020 | 0.199* |  | 0.538 | 0.053 |
| The sum of stigma and style length | 0.097 | 0.143 |  | 0.042 | 0.175* |  | 0.194 | 0.112 |

* indicated significant at α=0.05 probability level.
