# Supplementary material for: SYL3-k increases style length and yield of F1 seeds via enhancement of endogenous GA4 content in Oryza sativa L. pistils
Source: Theor Appl Genet. 2021 Oct 17;135(1):321–36. doi: 10.1007/s00122-021-03968-y (PMC8741667; doi:10.1007/s00122-021-03968-y)
Supplement: Supplementary file 7 — Supplementary file7 (DOCX 16 KB) [file 122_2021_3968_MOESM7_ESM.docx]

**Table S6** The SNP distribution in the CDS sequence of *SYL3* gene locus of 108 male sterile lines

| Material name | Type | SNPs | | |  | Material name | Type | SNPs | | |
| --- | --- | --- | --- | --- | --- | --- | --- | --- | --- | --- |
|  |  | S1 | S2 | S3 |  |  |  | S1 | S2 | S3 |
| Xiangling750S | Indica | G | G | G |  | Shenhu9A | Japonica | G | G | G |
| Yin28S | Indica | G | A | G |  | Huhan21A | Indica | A | G | G |
| Yuan15S | Indica | G | G | G |  | Huhan39A | Indica | A | G | G |
| Peiai64S | Indica | G | G | A |  | 212A | Indica | G | G | G |
| 01A | Japonica | G | G | G |  | Guang8A | Indica | G | G | G |
| Lvfeng009S | Indica | G | G | G |  | GuangheA | Indica | G | G | G |
| MingS | Indica | G | G | G |  | Shen9A | Indica | G | G | G |
| Shen08S | Indica | A | G | G |  | Ling30A | Indica | G | G | A |
| Lv102S | Indica | A | G | A |  | Ling27A | Indica | G | G | A |
| WangS | Indica | A | G | A |  | Q6A | Indica | G | G | A |
| Xiangling628S | Indica | A | G | G |  | Q4A | Indica | G | G | G |
| XinhuaS | Indica | A | G | G |  | Q2A | Indica | G | G | G |
| GuangtaiA | Indica | G | G | G |  | Q1A | Indica | G | G | G |
| YexiangA | Indica | G | G | G |  | 859A | Indica | G | G | G |
| 1892S | Indica | G | G | G |  | 813A | Indica | G | G | G |
| 9201A | Japonica | G | G | G |  | 817A | Indica | A | G | G |
| Zhendao88A | Japonica | G | G | G |  | ZhenyeA | Indica | A | G | G |
| Xu2A | Japonica | G | G | G |  | Zhennong257A | Indica | A | G | G |
| 6427A | Japonica | G | G | G |  | Jing4155S | Indica | G | G | G |
| 863A | Japonica | G | G | G |  | Longke638S | Indica | G | G | G |
| 9522A | Japonica | G | G | G |  | K186S | Indica | G | G | G |
| Zhenshan97A | Indica | A | G | G |  | RGD7S | Indica | G | G | G |
| LongtepuA | Indica | G | G | G |  | 9311S | Indica | G | G | G |
| ZhenpinA | Indica | A | G | G |  | DS | Japonica | G | G | G |
| Yun99S | Indica | G | G | G |  | ZhenxiangS | Indica | G | G | G |
| Y58S | Indica | A | G | G |  | Quanxiang9A | Indica | G | G | G |
| Z913S | Indica | G | G | G |  | QuanzaoA | Indica | G | G | G |
| ChengS | Indica | G | G | A |  | TaifengA | Indica | A | G | G |
| Chuang5S | Indica | G | G | G |  | WuxiangA | Indica | A | G | G |
| Feng99S | Indica | G | G | G |  | Wan9A | Indica | G | G | G |
| FuS | Indica | G | G | A |  | Huhan2S | Japonica | G | G | G |
| Guangxiang24S | Indica | G | G | G |  | Huhan9S | Indica | G | G | A |
| Guangzhan63S | Indica | G | G | G |  | Huhan23S | Indica | G | G | A |
| HanS | Indica | G | G | G |  | EK2S | Indica | A | G | G |
| HualianS | Indica | G | A | G |  | EK3S | Indica | A | G | G |
| KeS | Indica | G | G | G |  | Enong1S | Indica | A | G | G |
| Yu805A | Indica | G | G | A |  | Enong2S | Indica | G | G | G |
| Yu802A | Indica | G | G | A |  | Jin7A | Indica | G | G | G |
| Yu650A | Indica | G | A | G |  | Quan9311A | Indica | G | G | G |
| Xinong1A | Indica | G | A | G |  | YuanxiangA | Indica | A | G | G |
| Wan73A | Indica | G | G | A |  | Shennong2A | Indica | G | G | G |
| Wan23A | Indica | G | G | A |  | Shennong4A | Indica | A | G | G |
| Xin8S | Japonica | G | G | G |  | Jin23A | Indica | G | G | G |
| Nongken58S | Japonica | G | G | G |  | Jin37A | Indica | G | G | A |
| 7001S | Japonica | G | G | G |  | JinxiangA | Indica | G | G | A |
| Zhennong256A | Indica | A | G | A |  | JingtaiA | Indica | A | G | A |
| YuetaiA | Indica | A | G | G |  | Jing1A | Japonica | G | G | G |
| 9522A | Japonica | G | G | G |  | LvsanA | Indica | G | G | G |
| Shen9A | Japonica | G | G | G |  | C815S | Indica | G | G | G |
| Sidao8haoA | Japonica | G | G | G |  | Lu18S | Indica | G | G | G |
| 10A | Japonica | G | G | G |  | Yun9S | Indica | G | G | G |
| 130A | Japonica | G | G | G |  | Zheke82S | Indica | A | G | G |
| 390A | Japonica | G | G | G |  | Zhonghua2S | Indica | G | G | A |
| HanfengA | Japonica | G | G | G |  | ZitaiS | Indica | G | G | G |
